# Sucrose Starvation Induces Microautophagy in Plant Root Cells

**DOI:** 10.3389/fpls.2019.01604

**Published:** 2019-12-03

**Authors:** Shino Goto-Yamada, Kazusato Oikawa, Jakub Bizan, Shuji Shigenobu, Katsushi Yamaguchi, Shoji Mano, Makoto Hayashi, Haruko Ueda, Ikuko Hara-Nishimura, Mikio Nishimura, Kenji Yamada

**Affiliations:** ^1^Malopolska Centre of Biotechnology, Jagiellonian University, Krakow, Poland; ^2^Department of Cell Biology, National Institute for Basic Biology, Okazaki, Japan; ^3^NIBB Core Research Facilities, National Institute for Basic Biology, Okazaki, Japan; ^4^Department of Basic Biology, SOKENDAI (The Graduate University for Advanced Studies), Okazaki, Japan; ^5^Department of Bioscience, Nagahama Institute of Bioscience and Technology, Nagahama, Japan; ^6^Faculty of Science and Engineering, Konan University, Kobe, Japan

**Keywords:** microautophagy, autophagy-related genes, sucrose starvation, tonoplast, vacuole, E-64d, FM4-64, microdomain

## Abstract

Autophagy is an essential system for degrading and recycling cellular components for survival during starvation conditions. Under sucrose starvation, application of a papain protease inhibitor E-64d to the *Arabidopsis* root and tobacco BY-2 cells induced the accumulation of vesicles, labeled with a fluorescent membrane marker FM4-64. The E-64d–induced vesicle accumulation was reduced in the mutant defective in autophagy-related genes *ATG2*, *ATG5*, and *ATG7*, suggesting autophagy is involved in the formation of these vesicles. To clarify the formation of these vesicles in detail, we monitored time-dependent changes of tonoplast, and vesicle accumulation in sucrose-starved cells. We found that these vesicles were derived from the tonoplast and produced by microautophagic process. The tonoplast proteins were excluded from the vesicles, suggesting that the vesicles are generated from specific membrane domains. Concanamycin A treatment in GFP-ATG8a transgenic plants showed that not all FM4-64–labeled vesicles, which were derived from the tonoplast, contained the ATG8a-containing structure. These results suggest that ATG8a may not always be necessary for microautophagy.

## Introduction

Autophagy is one of the cellular degradation systems in eukaryotes that removes unwanted or toxic cellular components. Among several autophagic pathways, macroautophagy is well-studied and widely known; the term “autophagy” refers to macroautophagy in many cases. In the macroautophagy, cytosolic components are surrounded by a double-layered isolation membrane to form an autophagosome. The isolated cytosolic components in the autophagosomes are transported to vacuoles (yeasts and plants) or fused to lysosomes (animals), and then degraded by enzymes in these lytic organelles (Ohsumi, 2001). Macroautophagy was initially identified as a starvation-induced response in yeast in which macroautophagy recycles carbon and nutrients to survive starvation (Takeshige et al., 1992). In the macroautophagy process occurring in starvation, cellular components are non-selectively engulfed by autophagosome membranes for degradation. On the other hand, numerous recent reports demonstrated the phenomenon of selective macroautophagy, which functions toward selected targets such as damaged organelles and aggregated proteins (Anding and Baehrecke, 2017). Many autophagy-related (*ATG*) genes have been identified from yeast studies. The homologous genes responsible for core macroautophagic machinery are functionally conserved in plants (Yoshimoto, 2012).

In the case of “micro”-autophagy, the cellular contents are directly taken up into vacuoles by protrusion or invagination of vacuolar or lysosomal membranes. This mechanism is well reported in yeast species (Muller et al., 2000; Uttenweiler et al., 2005; Oku and Sakai, 2018). Recent studies demonstrated the existence of microautophagy in mammalian cells, and its ability to directly engulf endosomes, lipid droplets, and organelles into the lysosome/vacuole. In *Arabidopsis*, anthocyanin aggregates in the cytosol are captured and transported into the vacuole by microautophagy. During this microautophagy process, anthocyanin aggregates are surrounded by protrusions of the vacuolar membrane (tonoplast) in an ATG-independent manner (Chanoca et al., 2015). Additionally, whole chloroplast degradation, namely chlorophagy, proceeds *via* microautophagy; damaged and swollen chloroplasts exposed to excess light are directly engulfed by the tonoplast. Unlike the case of anthocyanin aggregates, this process requires *ATG5* and *ATG7* genes (Nakamura et al., 2018). Thus, the importance of ATGs in microautophagy is unclear and is therefore necessary to address in more detail.

As mentioned above, autophagy serves in removing anomalies and maintaining cell homeostasis in plants, as well as yeasts and mammals. Abnormal chloroplasts and dysfunctional mitochondria are the targets for autophagy, and a part of the endoplasmic reticulum (ER) is discarded *via* autophagy during ER stress (Liu et al., 2012; Broda et al., 2018; Nakamura et al., 2018). We have found that autophagy is also responsible for peroxisome quality control. Peroxisomes are ubiquitous organelles that are found in eukaryotic cells. We isolated *peroxisome unusual positioning* (*peup*) mutants which have an excess number of peroxisomes, and found that *peup1*, *peup2*, and *peup4* mutants have a defect in *ATG2*, *ATG18a*, and *ATG7*, respectively (Shibata et al., 2013). In these mutants, peroxisomes are more oxidized compared with that in the wild-type plants. Because several oxidases, such as acyl-CoA oxidases and glycolate oxidase, are involved in peroxisomal metabolic pathways, hydrogen peroxide is produced as a by-product of these enzymes in peroxisomes (Nishimura et al., 1983; Kirsch et al., 1986; Schrader and Fahimi, 2006). Due to hydrogen peroxide, peroxisomes are continuously oxidized and damaged. Our study indicated that highly oxidized whole peroxisomes are eliminated by autophagy, namely pexophagy, although some damaged peroxisomal proteins are maintained by chaperone function of peroxisomal Lon protease 2 (LON2) (Shibata et al., 2013; Goto-Yamada et al., 2014). The increased number of peroxisomes in the *peup* mutants is caused by the defect of autophagy. Therefore, we expected that the rest of the *peup* mutants, which also show excess peroxisomes, were also defective in autophagy/pexophagy.

To identify the genes that are involved in autophagy/pexophagy, we analyzed new *peup* mutants and determined the causative genes by whole-genome sequencing combined with map-based cloning. During this procedure, we used the rapid and straightforward determination of autophagy mutants; the absence of the aggregation of vesicles formed in root tip cells, which are induced by E-64d, which is an inhibitor for papain family protease (e.g. papain, cathepsin and, calpain), and visualized with FM4-64 dye. FM4-64 is a useful dye to visualize tonoplast; FM4-64 stains the plasma membrane passes through endosomes and then stains the tonoplast (Vida and Emr, 1995; Bolte et al., 2004). Previously, we reported that applying E-64d with FM4-64 to BY-2 cells and *Arabidopsis* roots induced the aggregation of FM4-64–stained vesicles besides the vacuole under starvation (Yamada et al., 2005). Moriyasu et al. reported that applying E-64d to BY-2 cells induced acidic vesicle aggregation (Moriyasu and Ohsumi, 1996). They also showed that applying E-64d to *Arabidopsis* root tips induced the aggregation of acidic compartments, which were stained with neutral red, and the formation of the aggregates of acidic vesicles was suppressed in the roots of *Arabidopsis atg2* and *atg5*, but it was not apparent in *atg9* (Inoue et al., 2006). Both BY-2 and *Arabidopsis* studies showed that sucrose starvation accelerated the formation of aggregates of both FM4-64–stained vesicles and acidic vesicles (Moriyasu and Ohsumi, 1996; Yamada et al., 2005; Inoue et al., 2006). Therefore, we expected that the vesicles stained with FM4-64 correlated to the acidic compartments and were related to autophagic machinery.

In this study, we first describe the procedure for identifying the causative genes in *peup17* and *peup22* mutants. Under starvation with the E-64d treatment, these mutants are defective in accumulation of vesicles in root cells. The *peup17* and *peup22* mutants are novel mutant alleles of *atg5* and *atg7*, respectively. In addition, we demonstrate that the vesicles are formed from the tonoplast, in a way similar to microautophagy, and that these vesicles do not contain tonoplast proteins. The vesicles capture cytosolic components and GFP-ATG8a–labeled autophagosomes, although not all vesicles inside the vacuole contain the ATG8a-containing structure. Our results provide further evidence that plants possess microautophagy and should improve our understanding on the fundamental processes of autophagy in plants.

## Materials and Methods

### Plant Materials and Growth Conditions

*Arabidopsis* (Columbia accession) and transgenic *Arabidopsis* expressing GFP in the peroxisome (GFP-PTS1) were used as the wild-type background (Mano et al., 2002; Mano et al., 2004). *Arabidopsis* mutants *peup1-1*, *peup2*, and *peup4* were also used (Shibata et al., 2013). T-DNA insertion mutants of *atg5-1* (SAIL_129B07, Thompson et al., 2005) and *atg7-2* (GK-655B06, Hofius et al., 2009) were obtained from the *Arabidopsis* Biological Resource Center (ABRC) and Nottingham *Arabidopsis* Stock Centre (NASC). The T-DNA insertions were confirmed by genome PCR using a gene-specific primer and a T-DNA primer as described in previous publications. The homozygous *ap2m-2* (SAIL_165_A05, Yamaoka et al., 2013) and *ap2s-1* (SALK_141555, Fan et al., 2013) mutants were provided by Dr. Shimada (Kyoto University, Japan). Organelle visualized lines, mGFP-VAMP713 and GFP-ARA7, and GFP-SYP43 were kindly provided from Dr. Ueda (NIBB, Japan) and Dr. Uemura (Ochanomizu University, Japan), respectively. 35Spro : GFP-ATG8a plants (N39996) were obtained from NASC (Thompson et al., 2005). To produce Venus-VAM3 transgenic plants, the Venus-VAM3/SYP22 pGWB1 plasmid (Ebine et al., 2008) was transformed into wild type Col-0 mediated by *Agrobacterium tumefaciens* (strain GV3101) using the floral dip method (Clough and Bent, 1998). All plants were germinated aseptically at 22°C under continuous light (∼100 µmol m^−2^ s^−1^) on 0.5× Murashige–Skoog (1/2 MS) growth media containing 0.4% (w/v) Gellan Gum (Wako, Tokyo, Japan), 0.5% (w/v) MES-KOH buffer (pH 5.7), 1% (w/v) sucrose, and 0.5× Murashige and Skoog salts mixture (Wako). For the sucrose-starvation test, sucrose was removed from the growth media.

### Identification of *PEUP17* and *PEUP22* Genes With Map-Based Cloning and Next-Generation Sequencing

The *peup17* and *peup22* (Col background) were crossed with Landsberg *erecta* (L*er*) to produce F1 and subsequently F2 progenies. A total of 47 or 20 F2 progenies expressing the *peup17* or *peup22* phenotypes, respectively, were scored according to their genetic background, as determined by a series of simple sequence length polymorphism (SSLP) markers (Bell and Ecker, 1994) which includes NARAMAP markers kindly provided by Dr. Tasaka (Nara Institute of Science and Technology, Japan) and Dr. Morita (National Institute for Basic Biology, Japan). Rough mapping located the *PEUP17* locus between the T9L3 and MPI7 BACs on chromosome 5, and the *PEUP22* locus between the marker NGA76 and the BAC MSN2 on chromosome 5.

To perform whole-genome sequencing, genomic DNA of *peup17* and *peup22* mutants were isolated using a DNeasy Plant Mini Kit (Qiagen). After shearing gDNA into 300–400 bp, library construction was performed as described in the TruSeq (Illumina, San Diego, CA, USA) manual. The libraries were sequenced on the HiSeq1500 (HO mode) with 101 bp paired-end reads. The sequenced data were mapped to the *Arabidopsis thaliana* genome reference TAIR10 with Bowtie2 (Langmead and Salzberg, 2012). Polymorphisms were called with SAMtools mpileup (Li et al., 2009) and filtered with a parameter varFilter -D100. Detected polymorphisms were annotated with SnpEff (Cingolani et al., 2012). The polymorphisms affecting amino acid sequence (non-synonymous mutations, stop-gained mutations, and mutations on splice donor/receptor sites) were searched in the regions restricted from the map-based cloning and 9 and 35 polymorphisms were listed in *peup17* and *peup22*, respectively.

Allelism tests were performed to confirm causative genes of *peup17* and *peup22* are *ATG5* and *ATG7*, each *peup* mutant was crossed with *atg5-1* and *atg7-2*, respectively, and the phenotypes of obtained F1 progenies were assessed.

### Chemicals and Fluorophores

FM4-64 and BCECF-AM were purchased from Thermo Fisher Scientific (CA, USA). E-64d and concanamycin A (ConA) were purchased from Sigma-Aldrich (MI, USA). These chemicals were prepared as stock solutions in dimethyl sulfoxide (DMSO). Quinacrine was purchased from Nacalai Tesque (Kyoto, Japan) and prepared as stock solutions in distilled water. The stock solutions (1.65 mM FM4-64, 1.13 mM BCECF-AM, 10 mM E-64d, 100 µM ConA, and 8 mM quinacrine) were stored at −20°C.

### Treatment of *Arabidopsis* Seedlings

**Figure 1:**
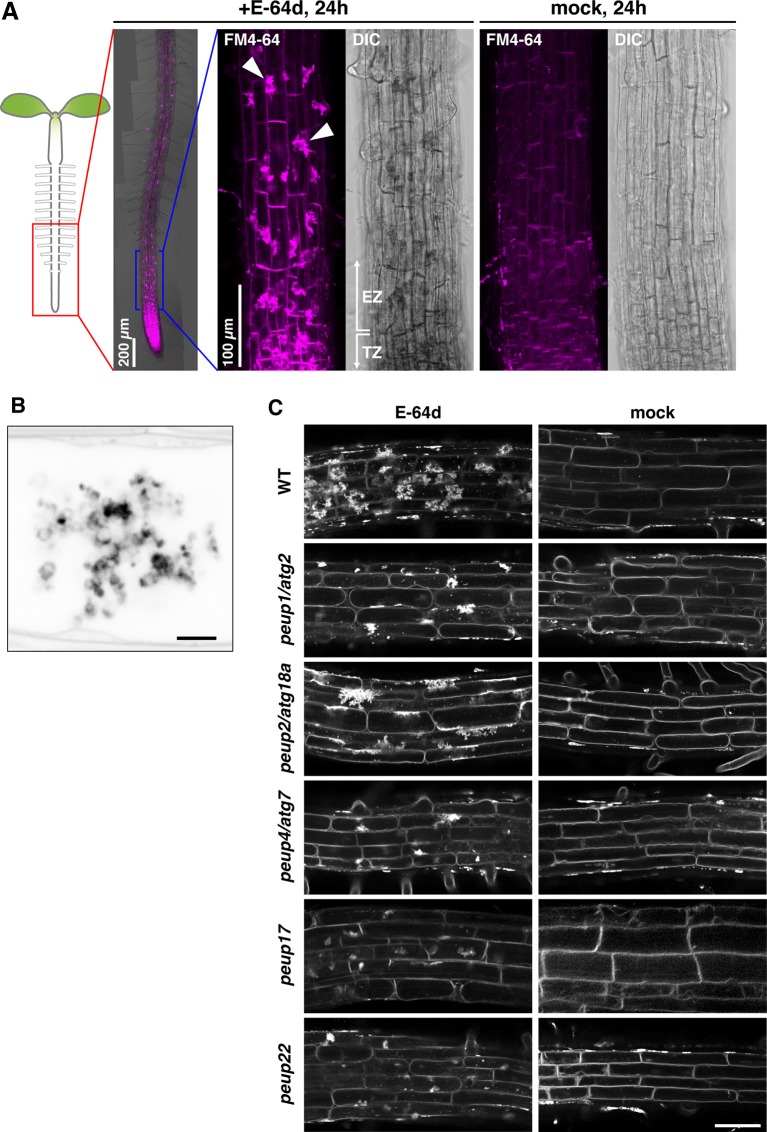
The phenotypes of E-64d vesicles in *peup* mutants. **(A)***Arabidopsis* roots were stained with FM4-64 with or without E-64d for 24 h in the wild-type (WT). TZ, transition zone; EZ, elongation zone. Arrowheads indicate the aggregates. **(B)** Magnified and inverted image of the vesicle aggregates stained with FM4-64. Bar = 2 µm. **(C)** The phenotypes of the accumulation of vesicles after the 24 h treatment of E-64d and FM4-64 in the WT and *peup* mutants. Bar = 50 µm.

**Figure 2:**
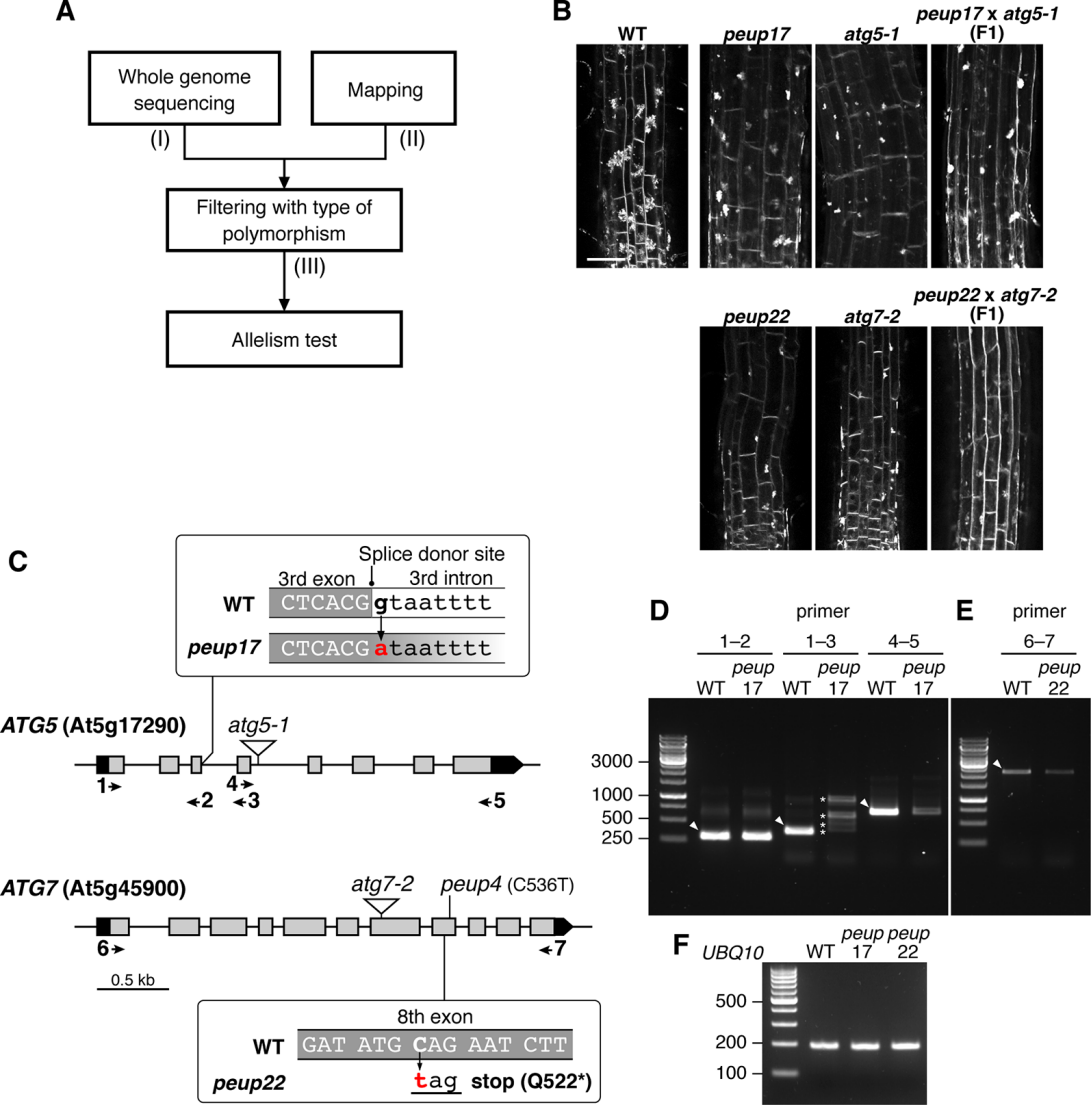
Identification of *PEUP17* and *PEUP22* genes. **(A)** A workflow of the identification of the causative genes in the *peup17* and *peup22* mutants. The information about (I) the number of polymorphisms detected with whole-genome sequencing, (II) the mapped region from map-based cloning, and (III) the number of candidate polymorphisms after filtering with the criteria of possibility to change the amino acid sequence are shown in Table 1. **(B)** Allelism test between *peup17* and *atg5-1* and *peup22* and *atg7-2*. The phenotype of E-64d vesicle formation was assessed in the root of the WT and F1 seedlings. The vesicles were stained with FM4-64 with E-64d for 24 h in the 6-day-old seedlings. Bar = 50 µm. **(C)** Schematic structure of *PEUP17/ATG5* and *PEUP22/ATG7* genes. The black and grey boxes indicate untranslated regions and exons, respectively. The *peup17* mutation substitutes the guanine to adenine at the splice-donor site in the third intron, and the *peup22* mutation substitutes the cytosine to thymine in the eighth exon and causes the substitution of glutamine at position 522 with a stop codon. The *peup4* is an allele of *peup22* (Shibata et al., 2013). The T-DNA insertion lines, *atg5-1* and *atg7-2*, are indicated by triangles. Arrows and numbers indicate the position of the primers used in following RT-PCR. **(D**–**F)** RT-PCR with cDNAs synthesized from the mRNAs of 7-day-old seedling of *peup17* and *peup22*. The positions of primers are indicated in **(C)**. **(F)** Indicates UBQ10 gene expressions. The sequence of the primers is in **Supplementary Table 1**. Arrowheads, expected size of bands in WT; asterisks, the unusual length of PCR products.

In **Figures 1**, **2**, and **4**, and **Supplementary Figures 3** and **5**, FM4-64 staining, E-64d treatment, and the induction of starvation were applied at the same time; 4- to 6-day-old seedlings grown on growth media are transferred to the FM4-64/E-64d solution (4 µM FM4-64 and 5 µM E-64d), and root cells were observed at 24 h. In other figures, seedlings were pre-stained before the treatment of inhibitors and the induction of starvation as shown below. Four- to 6-day-old *Arabidopsis* seedlings grown on growth media were transferred to the liquid 1/2 MS media containing 1% (w/v) sucrose [1/2 MS (+suc)] and 4 µM FM4-64. The seedlings in solution were covered by tin foil and kept at 22°C in the dark for 6–16 h. Then, FM4-64 dye was washed out with the incubation in 1/2 MS (+suc) for 2 h to stain only the tonoplast. The seedlings were briefly rinsed with the 1/2 MS media without sucrose [1/2 MS (−suc)] and transferred to the new 1/2 MS (−suc) to induce starvation concomitant with 5 µM E-64d or 0.5 µM ConA applying. For the mock treatment, DMSO was used instead of E-64d and ConA. After treatment, seedlings were mounted on glass slides and observed under a confocal microscope.

**Figure 3:**
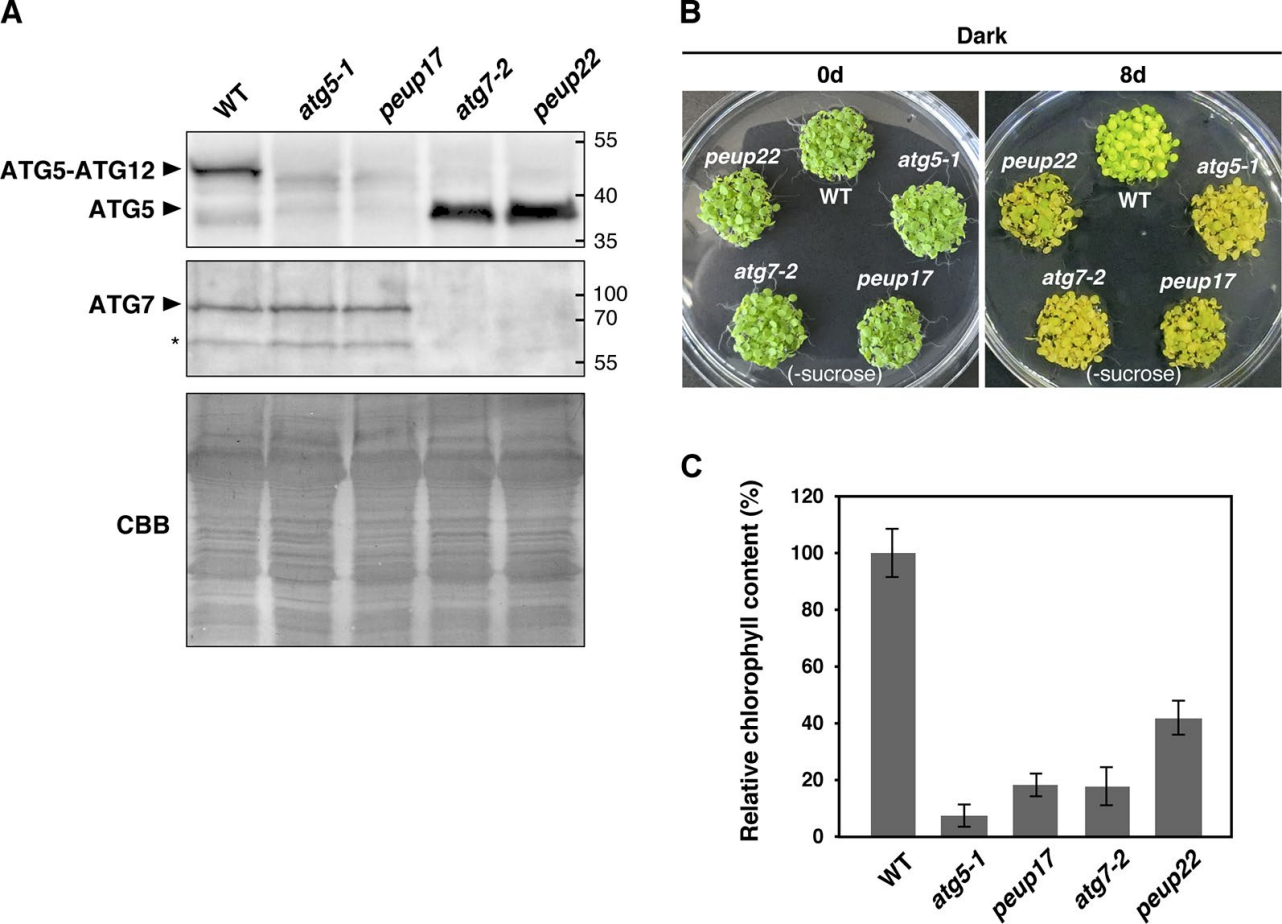
autophagy-related phenotypes in the *peup* mutants. **(A)** Immunoblot analysis of the *peup* and *atg* mutants. Crude protein extracts of the WT, *atg5-1*, *peup17*, *atg7-2*, and *peup22* from 7-day-old seedlings were subjected to SDS-PAGE and immunoblot analysis with anti-ATG5, and anti-ATG7, antibodies. Equal protein loading was confirmed by immunoblot analysis with Coomassie Brilliant Blue (CBB) staining. An asterisk indicates unknown bands specific to the anti-ATG7 antibody. **(B)** Senescence phenotypes of the *peup* and *atg* mutants. *Arabidopsis* seedlings were grown on sucrose deprivation media for 7 days and transferred to the darkness for 8 days. Photos were taken before and after 8 days of dark treatment and carbon deprivation. **(C)** Chlorophyll content in the WT and mutants. Chlorophylls were extracted from five seedlings, and six biological repeats were prepared for each plant lines. Chlorophyll content in WT was set as 1.0. Bar = ± SE, n = 6.

**Figure 4:**
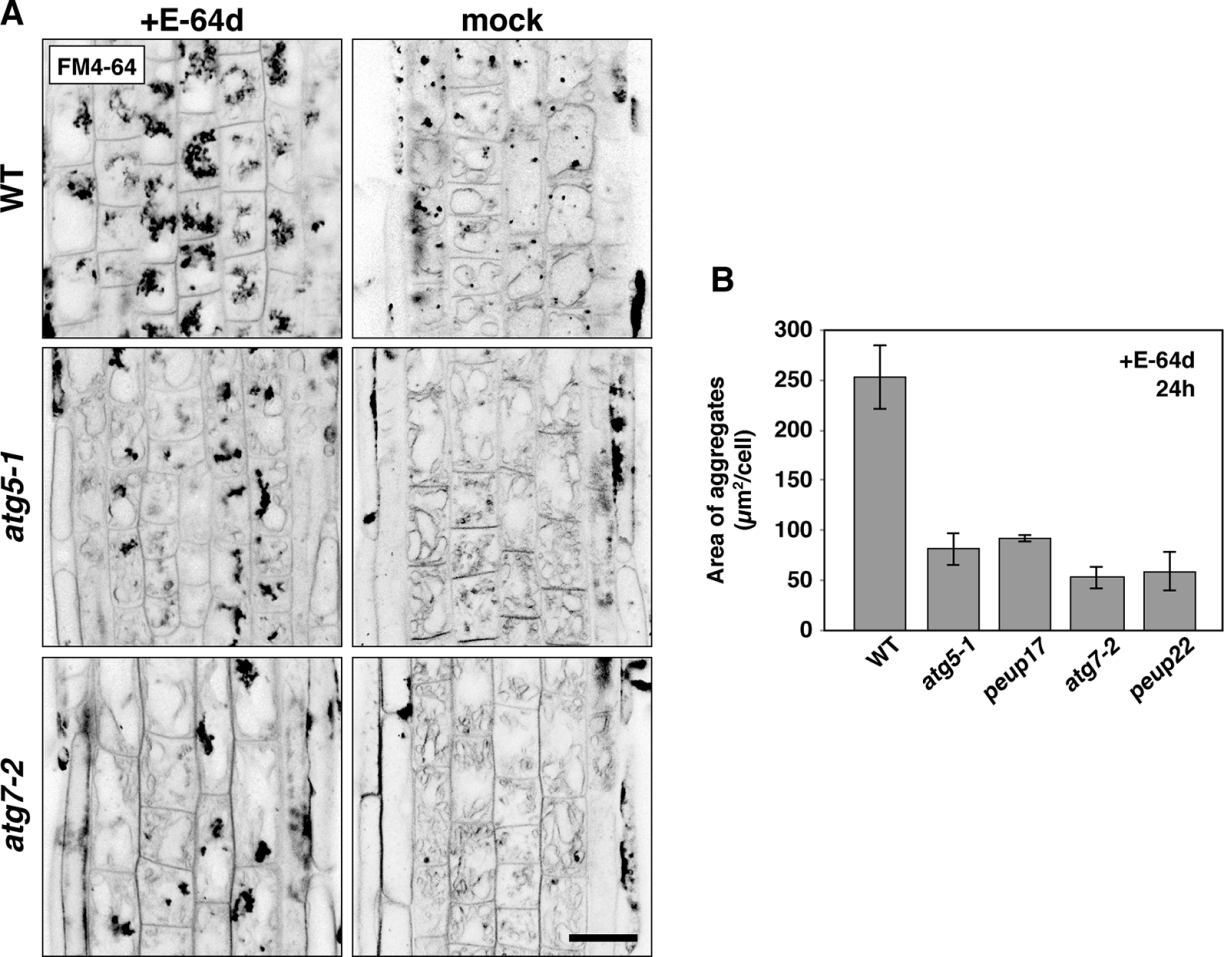
The phenotypes in E-64d vesicle formation in WT and *atg* mutants. **(A)** Inverted confocal micrographs of FM4-64 signals from 6-day-old seedlings treated with or without E-64d for 24 h under starvation. Images of the wide area from three replicates were shown in **Supplementary Figure 5**. Bar = 20 µm. **(B)** Quantification analysis of the area of aggregates in the WT and mutants upon E-64d treatment for 24 h. The area size per cell (µm^2^/cell) was obtained from ImageJ software. For making the graph, 8–10 cells from the transition zone were used in each plant, and three plants were used for each line. Bar = ± SE.

### Treatment of Tobacco Culture Cells

Suspension-cultured cells of tobacco BY-2 (*Nicotiana tabacum* cv Bright Yellow 2) were cultured in MS medium with 3% (w/v) (87.6 mM) sucrose in an orbital shaker at 140 rpm and 26°C in the dark. The cells were transferred to new medium at 1-week intervals.

Tonoplasts and E-64d vesicles were visualized essentially as described by Yamada et al. (2005). To stain tonoplasts with FM4-64, 3-day-old cells were incubated with culture medium containing 8 µM of FM4-64 for 1 day. After confirming that all FM4-64 are observed tonoplast under a microscope, the cells were collected by centrifugation at 100 × *g* for 2 min. The cell pellets were resuspended in new MS medium containing 87.6 mM of mannitol and no sucrose (sucrose-free medium). After an additional centrifugation step, the cells were suspended in two volumes of the original culture medium. E-64d (10 µM) was added to 1 ml of cell suspension in a 50 ml Falcon tube. ConA (100 nM) was added to inhibit V-ATPase. The culture was shaken at 140 rpm on an orbital shaker at 26°C in the dark. After the incubation, we added quinacrine (1.6 mM) or BCECF-AM (6 µM) to the culture medium to stain vacuoles and acid vesicles. After 5 min, the cells were washed several times with 5 mM HEPES-Na (pH 7.5) containing 0.1 M sorbitol and then observed under a confocal microscope. The number of BCECF-stained acid granules were counted from more than 15 cells on confocal images and graphed in **Supplementary Figure 6**.

### Confocal Laser-Scanning Microscopy

*Arabidopsis* seedlings and leaves were observed with confocal laser scanning microscopes (LSM880, Carl Zeiss). The GFP signal was observed by excitation with an Argon laser at 488 nm and detection between 493 and 598 nm. Chlorophyll signal was detected by excitation with a HeNe laser at 633 nm and detection at 638–722 nm. The FM4-64 signal was observed by excitation with a 514 nm Argon laser and detection at 592–759 nm. For simultaneous detection of FM4-64 and Venus, the 514 nm laser was used for the excitation and detected between 519 and 573 nm and 698 and 759 nm for Venus and FM4-64, respectively. For measuring the area of aggregates of E-64d vesicles, we used ImageJ software (https://imagej.nih.gov/ij/). For making the graph, 8–10 cells from the transition zone were used in each plant, and three plants were used for each line.

The double-stained BY-2 cells (FM4-64 and BCECF-AM or quinacrine) were observed with an Axiophoto microscope (Carl Zeiss, Jena, Germany) equipped with a confocal laser-scanning microscopy unit (CSU10/UZ, Yokogawa, Japan), a 488 nm LED laser (Sapphire 488-20, Coherent, Santa Clara, CA), and the appropriate filter set. Images were acquired with a CCD camera (DXM1200, Nikon, Japan).

### RT-PCR

Total RNAs were isolated from *Arabidopsis* seedlings with Trizol (Thermo Fisher Scientific) and 1 µg of total RNA was used to synthesize first-strand cDNA with Ready-to-Go RT-PCR beads (GE Healthcare, USA) and poly dT primers. The cDNA was used as a template of the PCR to amplify a part or a whole sequence of ATG5 or ATG7 cDNA with specific primer sets (**Supplementary Table 1**). PCR products were separated by agarose gel electrophoresis.

### Immunoblot Analysis

*Arabidopsis* seedlings were homogenized with extraction buffer containing 20 mM Tris-HCl, pH 6.8, 10% (v/v) β-mercaptoethanol, 2% (w/v) SDS, and 24% (v/v) glycerol. Homogenates were centrifuged at 20,000 × *g* for 10 min, and supernatants were boiled at 95°C for 5 min. Proteins were separated with SDS-PAGE and transferred to a polyvinylidene fluoride membrane (Millipore, USA) in a semidry electroblotting system. The membranes were subjected to immunoblot analysis using anti-ATG5 (AS15 3060, Agrisera, 1:5,000 dilution), and anti-ATG7 (AS15 3061, Agrisera, 1:5,000 dilution) with Can Get Signal solution (TOYOBO, Japan). Immunoreactive bands were detected by monitoring the activity of a horseradish peroxidase–coupled antibody against rabbit IgG (G-21234, Thermo Fisher Scientific, USA) and SuperSignal West Femto (Thermo Fisher Scientific).

### Chlorophyll Measurement

Chlorophyll contents were measured as described by Porra et al. (1989). Total chlorophylls were extracted from the same amount of frozen powdered samples with N,N-Dimethylformamide (DMF). Chlorophyll amounts were measured with 647 and 664 nm using the spectrophotometer. Chlorophylls were extracted from five seedlings in one experiment, and six biological repeats were prepared.

### Measurement of the Fluorescence Intensity

Vesicles in the vacuole were selected with the command Analyze Particle in ImageJ software. Then, GFP-ATG8a fluorescence was measured in the FM4-64–labeled vesicles. Autophagosomes in the cytosol were directly selected, and the intensities were measured. For the measurement of cytosolic intensity, three to five points were randomly chosen from the cytosolic area. The mean of these cytosolic intensities was used for normalization of signal intensity in a cell. Bars above the plots indicate mean ± SD, and the mean and standard deviations were calculated based on binary logarithm transformation. Total 23 cells from more than six plants were analyzed.

### Accession Numbers

Sequence data from this article can be found in the *Arabidopsis* Genome Initiative databases under the following accession numbers: At3g19190 (PEUP1/ATG2), At3g62770 (PEUP2/ATG18a), At5g45900 (PEUP4/PEUP22/ATG7), At5g17290 (PEUP17/ATG5), At4g21980 (ATG8a), At5g46630 (AP2M), At1g47830 (AP2S), At5g46860 (VAM3/SYP22), At3g05710 (SYP43), At4g19640 (ARA7), and At5g11150 (VAMP713).

## Results

### The *peup17 and peup22* Mutants, as Well as the *atg* Mutants, Show Defects in Accumulation of E-64d Vesicles

We have previously shown that the *peup* mutants were isolated from the ethylmethane sulfonate (EMS)–mutagenized parental plants that express a peroxisome marker GFP-PTS1 in the background of *Arabidopsis* accession Columbia (Mano et al., 2002; Shibata et al., 2013). These mutants were classified into several phenotypic groups. Both *peup17* and *peup22* mutants have an excess number of peroxisomes in leaves like *peup1/atg2*, *peup2/atg18a*, and *peup4/atg7* that are defective in autophagy machinery (**Supplementary Figure 1**, Shibata et al., 2013). Therefore, we expected that new *peup* mutants would have autophagy defects as well.

The wild-type root formed a huge aggregate in each cell after treatment with E-64d and FM4-64 under sucrose starvation for 24 h; the formation of aggregates was as hard at the tip of the root and moderate toward the top, and the mock treatment with FM4-64 rarely produced aggregates in the root tip (**Figure 1A**). These aggregates were composed of many vesicles (**Figure 1B**). In the *peup1/atg2* and *peup4/atg7* mutants, the accumulation of E-64d–induced vesicles (hereafter called E-64d vesicles) was lower than that observed in the wild-type plants (**Figure 1C**). To assess whether E-64d and FM4-64 treatment was able to distinguish new *peup* mutants from wild-type plants, we treated *peup17* and *peup22* mutants with the compounds and observed the phenotype. Similar to *peup1* and *peup4*, the suppression of aggregates was observed in *peup17* and *peup22* (**Figure 1C**). The *peup2/atg18a* roots formed the aggregates of E-64d vesicles, although the number of vesicles was fewer than those in the wild-type (**Figure 1C**). To verify whether the vesicles are peroxisome-related structures, we observed the subcellular localization of peroxisomes and E64d vesicles in root cells. Peroxisomes were neither localized at E64d vesicles nor the vesicle aggregates (**Supplementary Figure 2**), suggesting that these vesicles are not related to peroxisome degradation in the root tip cells. To assess whether the formation of E-64d vesicles is related to clathrin-dependent endocytosis, we treated the *ap2m-2* and *ap2s-1* mutants, which are defective in the components of the AP2 clathrin adaptor complex, µ- and σ-subunits, respectively (**Supplementary Figure 3**, Fan et al., 2013; Yamaoka et al., 2013). In these mutant roots, the aggregates of E-64d vesicles were formed as well as in the wild-type root, indicating that E-64d vesicles are formed *via* clathrin-independent pathways.

### Whole-Genome Sequencing and Map-Based Cloning Identified *peup17* and *peup22* as a New Allele of the *atg5* and *atg7* Mutants

We used E-64d vesicle phenotypes to rapidly and straightforwardly judge the mutant phenotype for the map-based determination of the responsible locus. We crossed the *peup* mutants with L*er* plants and separated mutant plants from the F2 progenies based on the phenotype upon E-64d and FM4-64 treatment. Then, we combined the map-based cloning and whole-genome sequencing to find the responsible genes in these mutants (**Figure 2A** and **Table 1**). The mapping identified the *PEUP17* locus in the 1.1 Mb length region on chromosome 5, which was mapped between the T9L3 and MPI7 bacterial artificial chromosome (BAC) clones using 47 F2 mutant plants. The locus of *PEUP22* was mapped to the 16.2 Mb length region on chromosome 5, which was between the marker NGA76 and the BAC MSN2, using 20 F2 mutant plants. Whole-genome sequencing revealed 9 and 35 polymorphisms in the mapped region in *peup17* and *peup22*, respectively, which were filtered with criteria of possibility to change the amino acid sequence (such as non-synonymous mutations, stop-gained mutations, and mutations on splice donor/receptor sites). The list of candidate genes of *PEUP17* and *PEUP22* included *ATG5* (At5g17290) and *ATG7* (At5g45900), respectively. To determine if *ATG5* and *ATG7* are causative genes of *peup17* and *peup22*, we produced F1 progenies by crossing *peup* mutants with T-DNA insertion mutants *atg5-1* (Thompson et al., 2005) and *atg7-2* (Hofius et al., 2009). Crossing between *peup17* and *atg5-1*, and *peup22* and *atg7-2* was not able to rescue the mutant phenotypes, both the failure to form aggregates of E-64d vesicles in root cells and the excess number of peroxisomes in leaves (**Figure 2B** and **Supplementary Figure 4**). These results demonstrated that *peup17* and *peup22* are new alleles of *atg5-1* and *atg7-2*, respectively.

**Table 1:** The information related to the determination of causative genes with whole-genome sequencing (WGS) and map-based cloning.

	(I) ^†^	(II) ^†^	(III) ^†^
	Polymorphisms identified with WGS^‡^	Mapped region (number of plants used in the mapping)	Polymorphisms affecting coding sequences in the mapped region
*peup17*	4,260	Chr 5: 4.8–5.9 Mb (47)	Non-synonymous: 7 Stop-gained: 1 Splice-site: 1
*peup22*	3,986	Chr 5: 10.4–26.6 Mb (20)	Non-synonymous: 32 Stop-gained: 2 Splice-site: 1

^†^ The numbers correlate to those in **Figure 2A**.

^‡^ The number of polymorphisms based on comparison with the parental plant.

The *peup17* mutation caused a substitution from guanine to adenine at the splice donor site in the third intron of the *ATG5* gene (**Figure 2C**). The mRNA from *ATG5* was assessed by reverse transcription and subsequent PCR (RT-PCR) with several sets of primers as shown in **Figure 2C** and **Supplementary Table 1**. We found RT-PCR products have varied lengths when the amplified region includes the third intron, in which the mutation has occurred at the splice donor site (**Figure 2D, F**). Moreover, the level of RT-PCR products was significantly reduced if the amplified region includes from the fourth exon to the last exon. These results indicated that the *peup17* mutation alters the splicing of *ATG5* mRNA to reduce the total amount of transcripts. The *peup22* mutation at the eighth exon of the *ATG7* gene resulted in the nonsense substitution of the glutamine in position 522 (**Figure 2C**). Although the *peup22* mutation was expected to affect the amino acid sequence, the amount of the RT-PCR product was decreased, suggesting that this mutation also alters the amount of *ATG7* mRNA (**Figure 2E, F**).

In the autophagy machinery, ubiquitin-fold proteins ATG8 and ATG12 are engaged for two ubiquitin-like conjugation systems acting in the isolation membrane formation. ATG12 is conjugated to ATG5 to produce ATG12–ATG5 conjugate (Geng and Klionsky, 2008), and the ATG7 protein is involved in conjugation cascades as an E1-like protein. To assess ATG7 function on the production of these conjugates in *peup17* and *peup22*, immunoblot analysis was performed (**Figure 3A**). In the ATG7 blotting, we detected two specific bands that disappeared in *atg7-2* and *peup22*. The major bands were slightly bigger than 70 kDa, which is the hypothetically expected size of ATG7. The minor band was around 65 kDa, probably the degradation product. In the *peup17* and *atg5-1* mutants, the band from ATG5 was not detected. The majority of ATG5 was detected as the ATG12–ATG5 conjugate in wild-type plants, whereas no conjugate was detected in *peup22* or in *atg7-2*, which is consistent with results of a previous report (Phillips et al., 2008). This result indicates that *peup22* is a null allele of *ATG7*.

To test the autophagic activity in *peup17* and *peup22*, we performed a carbon-deprivation test. One-week-old seedlings grown under the light were transferred into the dark, where photosynthesis is repressed and the plants start recycling their cellular components *via* autophagy to survive (Phillips et al., 2008; Yoshimoto and Ohsumi, 2018). Wild-type plants survived after 8-day dark treatment on the growth media lacking sucrose. On the other hand, *peup17* and *peup22* mutants failed to survive after the same condition (**Figure 3B**), and chlorophyll contents were much lower than that in wild-type plants (**Figure 3C**). These results indicate that autophagy under carbon starvation is suppressed in *peup17* and *peup22* as well as the mutants defective in *ATG5* and *ATG7* (Phillips et al., 2008).

### The *atg5* and *atg7* Mutants Failed to Form E-64d Vesicles and Increased the Amount of Tonoplast

As mentioned above, E-64d treatment induces the accumulation of many vesicles depending on autophagy (**Figures 1A and B**; Moriyasu and Ohsumi, 1996; Yamada et al., 2005; Inoue et al., 2006). However, the details of how these E-64d vesicles were formed and accumulated were not elucidated. After 24 h of E-64d treatment, E-64d vesicles were induced and formed aggregates. Without E-64d, similar vesicle aggregates are also visualized for 24 h, but they were much smaller than those upon the E-64d treatment (**Figure 4A**). This indicates the vesicles are naturally formed without E-64d treatment, but they continually accumulate when E-64d is treated. In the *atg5-1* and *atg7-2* mutants, aggregates of E-64d vesicles formed but were more modest than that in wild-type, and it was the same in the *peup17* and *peup22* mutants (**Figures 4A, B** and **Supplementary Figure 5**). The aggregates rarely formed in these mutants in the absence of E-64d. Interestingly, the shape of wild-type vacuoles was smooth, but vacuole shapes in the mutants were highly complicated, which was even more pronounced in the absence of E-64d. The cells treated with E-64d had less tonoplast than cells without E-64d treatment, implicating that the tonoplast was taken up into the E-64d vesicles.

### Starvation-Induced Acid Granules Are Captured by the Tubular Invagination of Tonoplast and Taken Into the Vacuole With the E-64d Vesicles in Tobacco BY-2 Cells

**Figure 5:**
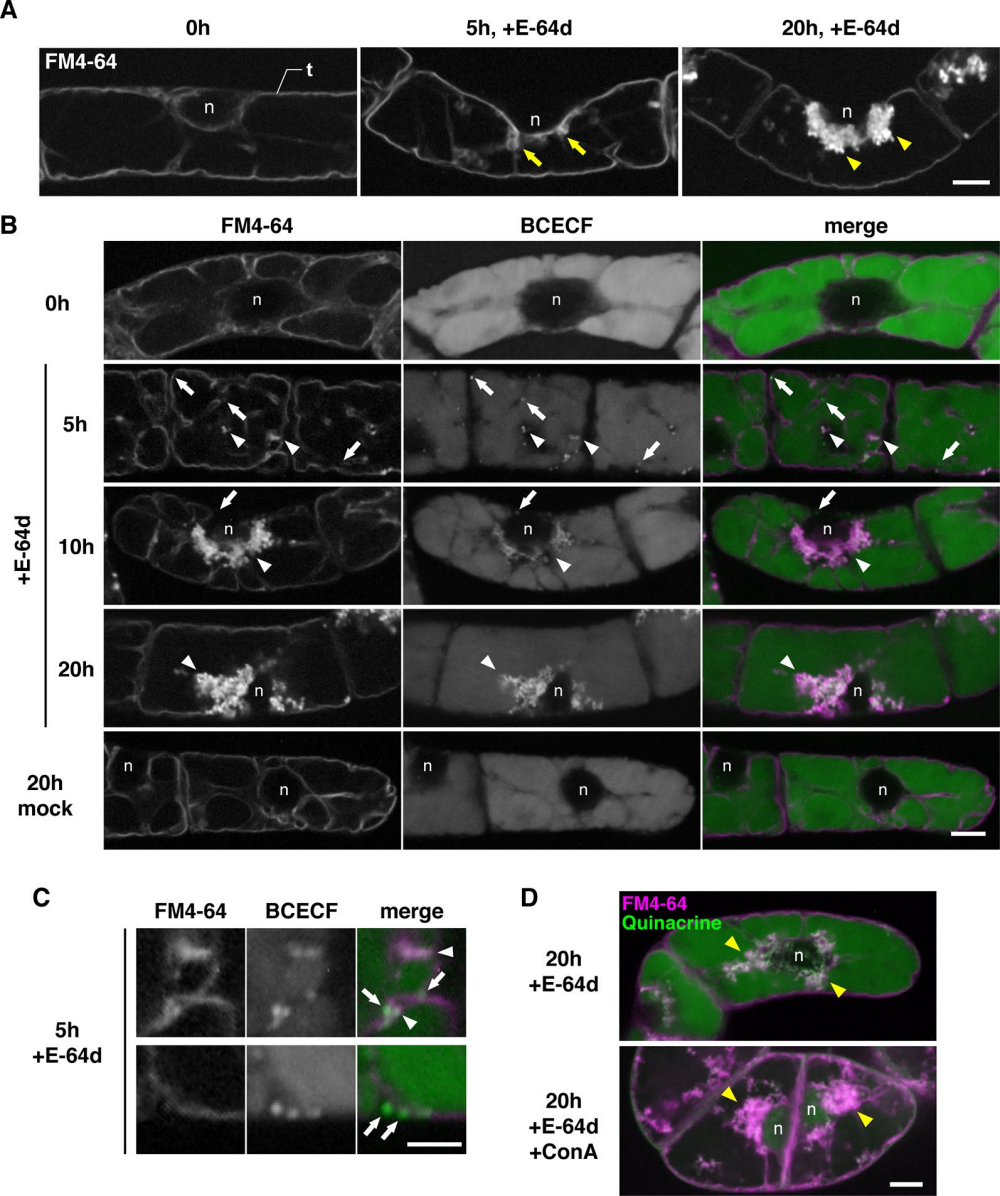
E-64d vesicles in sucrose-starved tobacco cells. **(A)** E-64d vesicles are generated from the tonoplast under starvation. The tonoplast was visualized with FM4-64 before the induction of starvation and E-64d treatment (left). Arrows indicate the invagination sites of tonoplast observed after 5 h of starvation and the treatment with E-64d (middle). Arrowheads indicate E-64d vesicles observed after 20 h of the treatment (right). Bar = 10 µm; n, nucleus; t, tonoplast. **(B)** Cytosolic acid granules are captured into E-64d vesicles. FM4-64 and BCECF staining show tonoplast and acidic compartments, respectively. Arrows indicate the cytosolic acid granules. Arrowheads indicate E-64d vesicles. Bar = 10 µm; n, nucleus. **(C)** High-magnification images showing that the cytosolic acid granules (arrows) are captured by tubular invagination of the tonoplast to form E-64d vesicles (arrowheads) after 5 h of treatment with E-64d. Bar = 5 µm. **(D)** Concanamycin A (ConA) treatment did not interfere with E-64d vesicle formation. Cells were treated with (upper panel) or without (lower panel) ConA. Magenta and green colors show FM4-64 and quinacrine fluorescence, respectively. Arrowheads indicate E-64d vesicles. Bar = 10 µm. n, nucleus.

To characterize the process of E-64d vesicle formation in detail, we used tobacco BY-2 cells, which is a model plant cell line. E-64d vesicle formation can be easily induced in tobacco BY-2 cells by exchanging culture media without sucrose. In addition, a population of uniform cell type facilitates the evaluation and observation of the effect of treatments. The tonoplast, but not the plasma membrane of the cells, was visualized by 1-day treatment of FM4-64 (**Figure 5A**, Yamada et al., 2005). After that, the cells were starved and treated with E-64d. Five hours after the starvation and E-64d treatment, cells started to form tubular structures on the tonoplasts (arrows in **Figure 5A**). After 20 h of treatment, E-64d vesicles had accumulated and formed large aggregates. These results were similar to the results from the *Arabidopsis* roots (**Figure 4**). We stained the cells with BCECF-AM to visualize vacuoles and acidic compartments (Matsuoka et al., 1997). After 5 h of starvation, cytosolic acid granules were observed before the formation of E-64d vesicles (arrows in **Figure 5B**, 5 h, and **Figure 5C**). Interestingly, some of the cytosolic acid granules were captured by the invaginated tubular structure of the tonoplast (arrowheads in **Figure 5B**, 5 h, and **Figure 5C**). Numerous acid granules colocalized with the aggregated E-64d vesicles at 10 h after treatment (arrowhead in **Figure 5B**, 10 h). After 20 h of the treatment, the induction of the acid granules had completed, and all granules localized at the aggregate of the E-64d vesicles (**Figure 5B**, 20 h). These results suggest that the cytosolic acid granules, which are induced by sucrose starvation, are taken up into the E-64d vesicles accumulated beside the tonoplast (**Supplementary Figure 6**). These acid granules would be assimilated into the vacuole, as no acid granules were observed in the vacuole in a mock treatment (**Figure 5B**, mock 20 h). Next, we examined whether vacuolar pH affects the process of E-64d vesicle formation. Treatment with ConA inhibited the acidification of the vacuole, which was represented by reduced quinacrine staining signals, but E-64d vesicles formed normally as shown in **Figure 5D**. This result suggests that the vacuolar pH does not affect the activity of the tonoplast invagination and the vesicle formation during sucrose starvation.

### E-64d Vesicles Are Derived From the Tonoplast but Do Not Include Tonoplast Proteins and Endosomes

**Figure 6:**
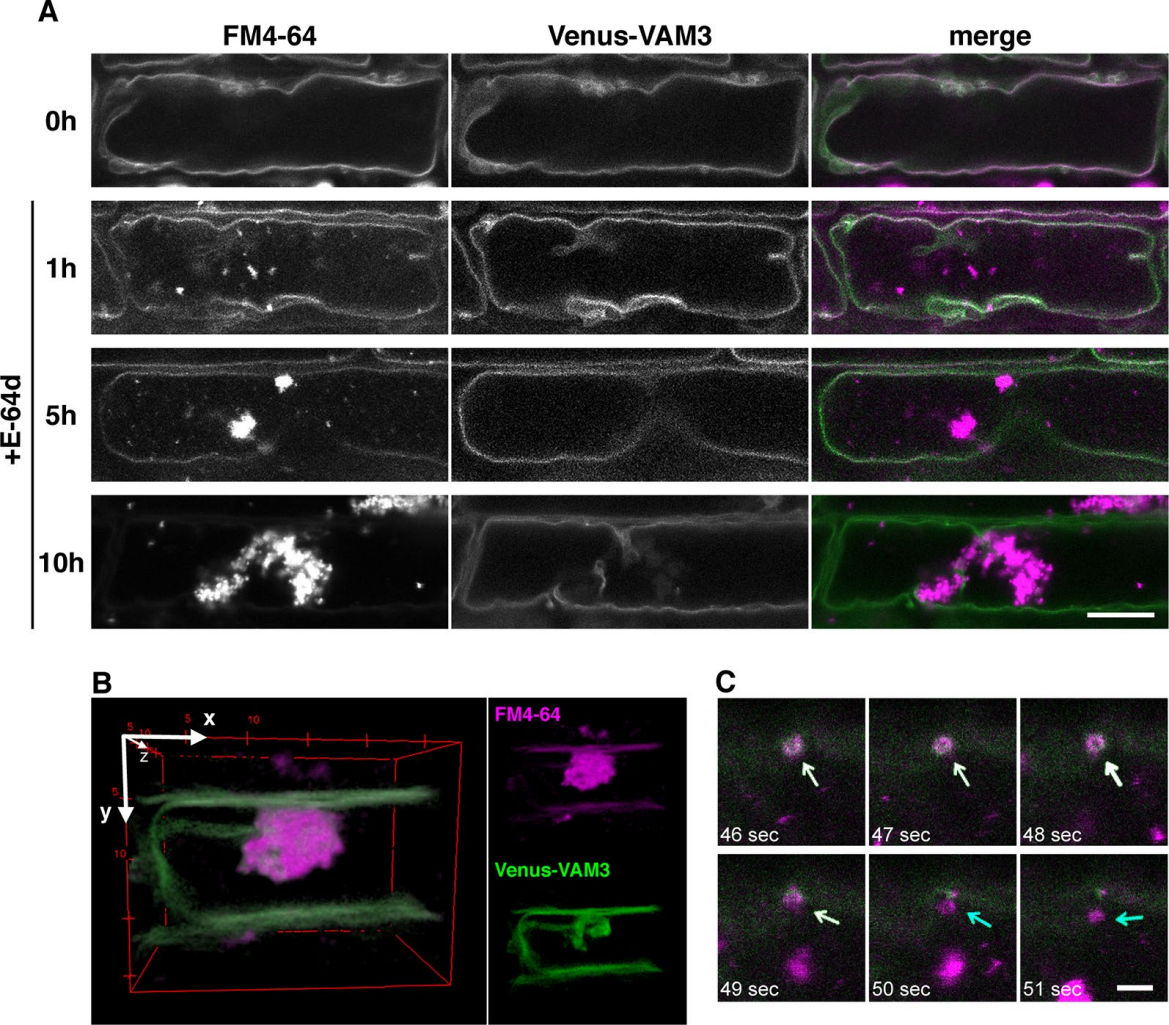
Simultaneous visualization of tonoplast protein and E-64d vesicles. **(A)** Confocal micrographs of root cells from Venus-VAM3 transgenic plants treated with E-64d under sucrose starvation. The tonoplast was visualized with FM4-64 before the induction of starvation and applying E-64d, and observed after 1, 5, and 10 h of the treatments. Magenta and green indicate the signals of FM4-64 and Venus, respectively. Bar = 10 µm. **(B)** Three-dimensional (3-D) image of the Venus-VAM3 root cell after 5 h of the treatments, which was reconstructed from a z-stack confocal microscopic images (0.3830 µm interval) with the ImageJ 3D Viewer plugin (https://imagej.nih.gov/ij/plugins/3d-viewer/). **(C)** An FM4-64–labeled vesicle is released from the tonoplast. The images were extracted from **Supplementary Movie 2**. The tonoplast in the Venus-VAM3 cell was pre-stained with FM4-64, and then cells were starved with E-64d treatment for 6 h. The elapsed time is indicated at the lower left corner of each image. White and blue arrows indicate the vesicles stained with both FM4-64 and Venus-VAM3 and only with FM4-64, respectively. Bar = 2 µm.

To clarify the relationship between the vacuolar membrane and the vesicle formation process, we used tonoplast-visualized transgenic plants. VAM3/SYP22 is a Syntaxin-related protein, which is located at the tonoplast and occasionally at prevacuolar compartments/late endosome (PVC/LE) (Ebine et al., 2008; Uemura et al., 2010). A plasmid designed to express a fusion of the fluorescent protein Venus and VAM3 under the control of VAM3 promoter (Ebine et al., 2008) was introduced into *Arabidopsis* plants to produce Venus-VAM3 transgenic plants. We treated Venus-VAM3 plants with FM4-64 and then washed out the dye by incubation with the growth media to stain only tonoplast. At this point, the FM4-64 signal was completely merged with the Venus-VAM3 signal on the tonoplast (**Figure 6A**, 0h). After the FM4-64 staining of the tonoplast, the plants were transferred to the growth media lacking sucrose, and concomitantly treated with the E-64d to induce the vesicle accumulation. One hour posttreatment, we recognized FM4-64 signals at the vesicle, and small aggregate structures on the tonoplast and inside the vacuole (**Figure 6A**, 1 h). At 5 h, the vesicle aggregates became evident, and the three-dimensional (3-D) view suggested that the aggregate was located inside the vacuole and closely associated to the transvacuolar strand (**Figure 6B** and **Supplementary Movie 1**). The time-lapse imaging captured that emerging vesicle, which was labeled with the Venus-VAM3 and FM4-64, and only FM4-64–labeled membrane was released from the tonoplast (**Figure 6C** and **Supplementary Movie 2**). The vesicles and small aggregates showed random motion, namely the Brownian motion, indicating the vacuolar localization of the vesicles (**Supplementary Movie 3**). Almost all FM4-64 signals were detected as vesicles which formed huge aggregate in the tonoplast at 10 h (**Figure 6A**, 5 and 10 h). Unlike the case at 0 h, the Venus-VAM3 signals were detected as discontinuous membranes and were not merged with the FM4-64 signals (**Figure 6A**, 10 h). Since *atg5-1* and *atg7-2* root cells form small aggregates of E-64d vesicles (**Figure 4**), we analyzed the subcellular localization of these vesicles in the mutant cells. The aggregates and small vesicles located in the vacuolar space and showed Brownian motion (**Supplementary Figure 7** and **Supplementary Movies 4** and **5**). These results indicate the vacuolar localization of E-64d vesicles in the mutant cells, although the numbers of vesicles are lesser than that of wild-type. Similar to the case in the BY-2 cells, vacuolar pH does not affect the E-64d vesicle formation from the tonoplast (**Supplementary Figure 8**).

To determine whether this phenomenon also occurred on other tonoplast proteins, we used the tonoplast marker line mGFP-VAMP713 plants which express mGFP-fusion of VAMP713 (Takemoto et al., 2018). The signal from mGFP-VAMP713 was merged with FM4-64–labeled membrane before starvation, whereas the signal was excluded from the E-64d vesicles after the induction of starvation, as well as Venus-VAM3 (**Supplementary Figure 9**). Next, we assessed the possibility that the endosomes formed the aggregates. The trans-Golgi network (TGN)/early endosome marker GFP-SYP43 plants (Uemura et al., 2012) and the late endosome marker GFP-ARA7 plants (Inada et al., 2016) were treated with E-64d under starvation. Both of GFP signals from GFP-SYP43 and GFP-ARA7 were detected on punctate structures in the cytosol, and absent on the E-64d vesicles (**Supplementary Figure 9**). These results supported that E-64d vesicles are not endosomes. The direct vesicle formation from the tonoplast is implicated in one of the autophagies, which directly incorporates cytoplasmic components into the vacuole by invagination of the vacuolar membrane, namely microautophagy (Muller et al., 2000; Oku and Sakai, 2018).

### The Tonoplast Captures Vesicles That Contain Cytoplasm and Autophagosomes

**Figure 7:**
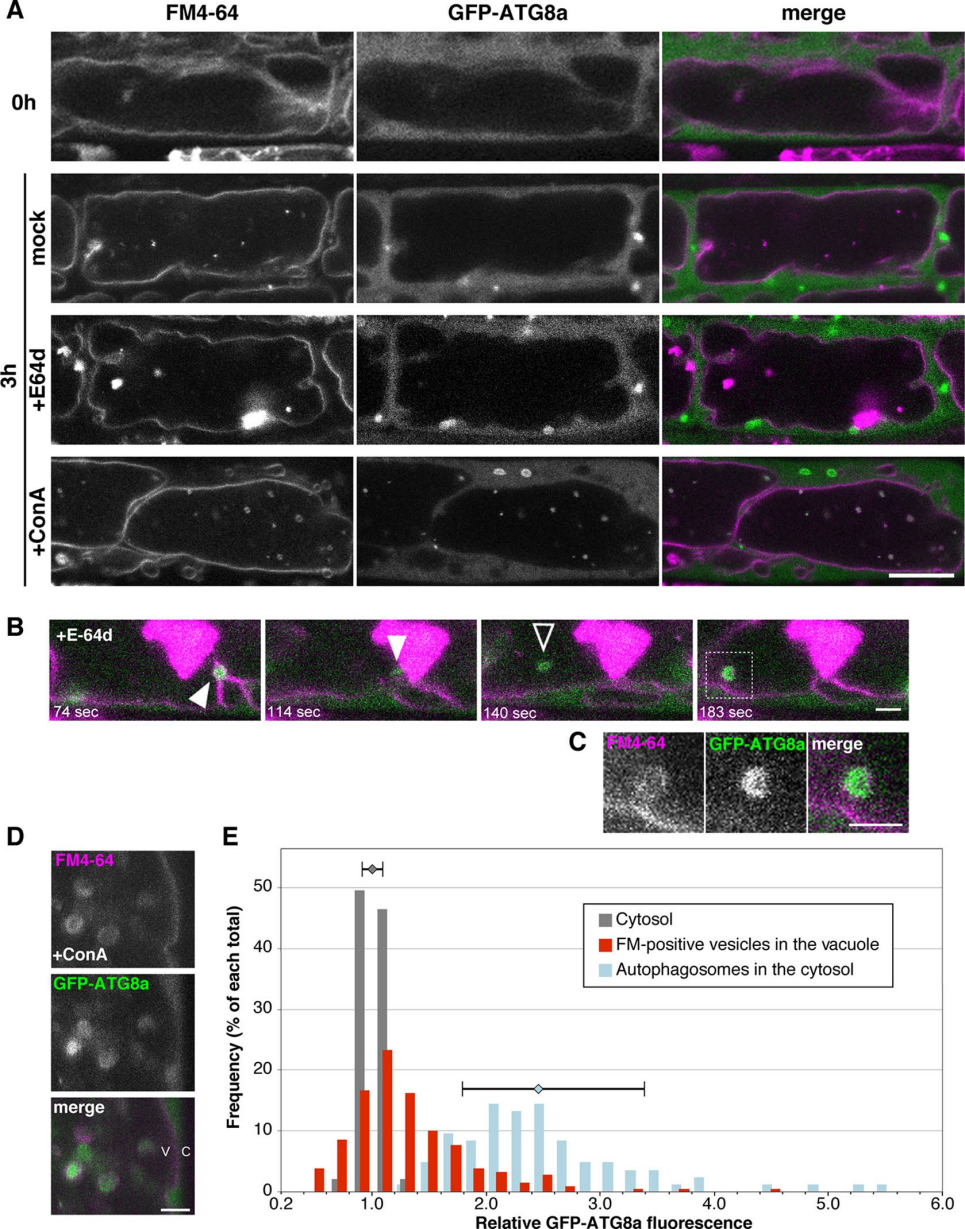
Subcellular localization of GFP-ATG8a during starvation and E-64d treatment. **(A)** Confocal micrographs of root cells from GFP-ATG8a transgenic plants treated with E-64d under sucrose starvation. The tonoplast was visualized with FM4-64 before the induction of starvation. Cells were starved and treated with E-64d or ConA for 3 h. Magenta and green indicate the signals of FM4-64 and GFP, respectively. Bar = 10 µm. **(B)** GFP-ATG8a–labeled cytosolic autophagosomes were trapped to the tonoplast and taken into the vacuole. The tonoplast in the GFP-ATG8a cell was pre-stained with FM4-64, and then cells were starved with E-64d treatment for 5 h. The images were extracted from **Supplementary Movie 6**. The elapsed time is indicated at the lower left corner of each image. White arrowheads indicate a captured autophagosome, and a black arrowhead indicates a vesicle which started random motion in the vacuole. Snapshot images of the longer time period are shown in **Supplementary Figure 10**. Bar = 2 µm. **(C)** Magnified image of the area indicated in the bottom image of **(B)** Bar = 2 µm. **(D)** Magnified images of a GFP-ATG8a cell treated with ConA under starvation for 3 h. Bar = 2 µm. v, vacuole; c, cytosol. **(E)** Histogram plots showing the profiles of the relative intensity of GFP-ATG8a of the cytosol (grey), FM4-64–labeled vesicles in the vacuole (red), and autophagosomes in the cytosol (blue) from the GFP-ATG8a root cells treated with ConA under starvation. Fluorescent intensities in the vesicles and the cytosol were measured with ImageJ and normalized with the mean of three to five points of cytosolic fluorescence in each cell. Frequency is shown in a percentage of each total of counts. Error bars show ± SD in the profiles of “cytosol” and “autophagosomes in the cytosol.” Total 23 cells from more than six plants were analyzed.

During degradation of a whole damaged chloroplast *via* microautophagy, namely chlorophagy, GFP-ATG8a–labeled structure accompanies the chloroplast engulfment by tonoplast (Nakamura et al., 2018). To clarify whether ATG8a is essential to microautophagy under sucrose starvation, we observed subcellular localization of GFP-ATG8a using 35Spro : GFP-ATG8a plants. Under the non-starved condition, the GFP-ATG8a signal was detected only at the cytosol (**Figure 7A**, 0 h). After induction of starvation, the strong GFP signal was detected as vesicle structures in the cytosol, which are expected to be autophagosomes as reported previously (Chen et al., 2017); these structures were rarely seen in the vacuole, whereas FM4-64–labeled structures were observed in the vacuole (**Figure 7A**, 3 h mock). Similarly, GFP-ATG8a signals were mainly located in the cytosol with the treatment of E-64d, and not colocalized with E-64d vesicles (**Figure 7A**, 3 h E-64d). Time-lapse images from the GFP-ATG8a root cells showed that GFP-ATG8a–labeled cytosolic autophagosomes were trapped to the tonoplast, and then shifted to the inside of the vacuole with the Brownian motion (**Figure 7B**, **Supplementary Figure 10**, and **Supplementary Movie 6**). These results implicate that autophagosomes could be taken by microautophagy and that multiple routes could be active under starvation in plat cells. It should be noted that at some times, we observed that the GFP-ATG8a signal vanished, and only the FM4-64 signal was left inside the vacuole (**Supplementary Movie 7**); this could be the reason GFP-ATG8a signal was not involved in the aggregates of E-64d vesicles. To keep vesicles with GFP fluorescence in the vacuole, we treated tonoplast-stained cells with ConA under starvation. Numerous GFP-ATG8a–labeled punctate structures were detected in the vacuole with ConA treatment, and most of the structures were co-labeled with FM4-64 (**Figure 7A**, 3 h ConA). As shown in the magnified image, the GFP-labels in the accumulated vesicles were varied (**Figures 7D, E**). A few vesicles had strong GFP signals comparable with the signal from autophagosomes in the cytosol, whereas the majority of vesicles had a low signal that was similar to that of the cytosol. This result indicates that not all vesicles contained GFP-ATG8a–labeled structure and the process of microautophagy is not always accompanied by the localization of ATG8a and/or ATG8-containing isolation membranes.

## Discussion

### Sucrose Starvation–Induced Microautophagy in Root Cells Involves a Part of Core ATG Proteins

Previously, we reported that treatment with E-64d produces vesicle aggregations, which is easily observed with FM4-64 dye in BY-2 cells and *Arabidopsis* root tips (Yamada et al., 2005). Moriyasu et al. reported that E-64d treatment induces aggregates of acidic vesicles, which is associated with autophagy, and electron microscope experiments showed that a large number of the vesicles were tethered at the interface of the cytosol and the vacuole; these vesicles contained acid phosphatases, which are recognized as a marker of lysosomes in mammalian cells (Moriyasu and Ohsumi, 1996; Inoue et al., 2006). We focused on this phenomenon as a tool to easily evaluate a large number of autophagy mutants. However, the detailed mechanism underlying this process and the site of the vesicle and the aggregate formation needed to be answered. In this study, we determined whether the acid vesicles were identical to the E-64d vesicles stained with FM4-64. Double staining with BCECF and FM4-64 in BY2 cells showed that tiny cytosolic acid granules were formed without FM4-64 staining, trapped into the invagination of the vacuolar membrane and then colocalized with FM4-64 signals. These results suggest that the E-64d vesicles became acidic by capturing the cytosolic acid granules which were observed with neutral red staining (Inoue et al., 2006).

We demonstrated that the E-64d vesicles are formed from the tonoplast, and vesicle formation is pronounced under the sucrose starvation. These results indicate that sucrose starvation induces “micro”-autophagy, which directly takes cytosolic components and membranes into the vacuole with protrusion or invagination of the vacuolar membrane/tonoplast (Oku and Sakai, 2018). Whether microautophagy requires ATG proteins seems to vary depending on the organism and system. In *Pichia pastoris*, unnecessary peroxisomes are directly engulfed by the vacuolar membrane, and several ATG proteins are required for this (Farre and Subramani, 2004). On the other hand, degradation of lipid droplets *via* a microautophagy-like system, called microlipophagy, does not require ATG7 but requires ESCRT components in *Saccharomyces cerevisiae* (Vevea et al., 2015). In plants, cytoplasmic anthocyanin aggregates contact the vacuolar surface and are surrounded by protrusions of the tonoplast, and the engulfment proceeds normally in the *atg5* mutant. On the other hand, elimination of membrane-damaged chloroplasts is arrested in the *atg5* mutant, in which engulfment of damaged chloroplasts is limited (Nakamura et al., 2018). In our study on plant root tips, microautophagy was induced under sucrose-starvation conditions, and ATG proteins were involved in the process; the mutants defective in ATG2, ATG5, and ATG7 showed the decreased number of microautophagic bodies. This phenomenon may be due to a deficiency in the vesicle formation on the tonoplast, because the amount of the tonoplast membrane was increased in the *atg5* and *atg7* mutants compared with that in the wild-type (**Figure 4A**). However, since autophagic body formation is not completely inhibited in these mutants, further analysis will be required to determine the importance of the roles of core ATGs in the microautophagy process. The *atg18a/peup2* mutant accumulates aggregates of E-64d vesicles (**Figure 1C**), and the accumulation of acidic vesicles was not effectively suppressed in the *atg9* mutant as well (Inoue et al., 2006), indicating the less contribution of these two genes in the microautophagy in the root cells under starvation. During the process of microautophagy, the damaged chloroplast is associated with ATG8-containing structures and engulfed by the tonoplast (Nakamura et al., 2018). We detected that GFP-ATG8a–labeled autophagosomes were formed in the cytosol under starvation and taken into the E-64d vesicles surrounding by tonoplast. However, not all FM4-64–labeled vesicles, which were remaining in the vacuole by ConA treatment, contain ATG8a-containing structure, and most of the vesicles showed cytosolic intensity of GFP fluorescence (**Figures 7A, D, and E**). Both of E-64d and ConA are reported to inhibit the degradation of autophagic cargos. E-64d inhibits protease function and suppresses cargo degradation after the fusion of autophagosomes and the lysosome/vacuole, whereas ConA inhibits the vacuolar type H+ATPase activity and inhibits acidification of the vacuole (Klionsky et al., 2016). While E-64d treatment causes vesicle aggregations, ConA-induced vesicles are scattered in the vacuole. The further discussion will be required to understand whether the vesicles formed by these two inhibitors have the same properties. However, because of the similarity between the vesicles from these two treatments (both vesicles are induced and accumulated in the vacuole under starvation, surrounded by a tonoplast-derived membrane, and the partial association of ATG8a-containing structure), we expected that these two phenomena are related. The results from the GFP-ATG8a cells with the ConA or E-64d treatment implicate that microautophagy induced under starvation mainly occurs without the association of ATG8-containing structure, although the possibility of involvement of other ATG8 isoforms needs to be verified in the future experiment.

### Is Microautophagy Initiated From a Specific Site on the Tonoplast?

FM4-64 is a dye that stains plasma membranes, endocytic compartments, and vacuolar membrane (Vida and Emr, 1995; Ueda et al., 2001). After 10 h of the E-64d treatment and starvation, the FM4-64 signal was mainly detected on the aggregated vesicles, and a few were detected on the entire tonoplast. These results indicate that the region that forms vesicles seems to have properties that are preferred by FM4-64 (**Figure 6**). A similar selectivity of the dye was observed in BY-2 cells; 1-day treatment of FM4-64 stained the whole vacuolar membrane, whereas upon the application of E-64d, the majority of dye was detected on the E64-d vesicles, and the signal in the tonoplast decreased (**Figure 5**). These phenomena point to the differences in membrane properties between the vesicle-forming region and the tonoplast.

FM dyes are taken into the plasma membrane just after their application, and prolonged incubation allows FM dyes to stain tonoplasts and nascent cell plates (Bolte et al., 2004). Additionally, the fungal macrocyclic lactone brefeldin A (BFA) treatment interrupts vesicle trafficking from the TGN toward the vacuole and FM dye accumulates at the BFA bodies (Kleine-Vehn et al., 2008). Thus, FM dyes strongly stain the location of the end of membrane trafficking. Under starvation conditions, the microautophagy degrades cellular components in the vacuole; the final location of E-64d vesicles should be the vacuole, but FM4-64 dye can be accumulated in the E-64d vesicles when the process was stopped. Interestingly, most of E-64d vesicles lack the tonoplast protein Venus-VAM3, although FM4-64 and Venus-VAM3 colocalized at the tonoplast early in E-64d vesicle formation (**Figure 6**). This is similar to the microdomains at the vacuolar membrane that occur in yeast during the course of microautophagy. Müller et al. reported that the tubular structures invaginated toward the vacuole lumen under nitrogen starvation in *S. cerevisiae*. Freeze-fracture analysis revealed that a restricted area that is not covered with tiny particles (large vacuolar-membrane proteins) exists at the vacuolar membrane, and the authors propose that this microdomain is the place of the early stages of autophagic tube formation (Muller et al., 2000). Tsuji et al. also suggested the contribution of microdomains in lipophagy, in which lipid droplets are taken into the vacuole *via* microautophagy in *S. cerevisiae*. During this process, raft-like domains are formed in the vacuolar membrane, which consist of specific lipids including sterols and sphingolipids. This microdomain is free from membrane proteins and engulf lipid droplets. ATG proteins may indirectly contribute to the process; sphingolipids are transported to the vacuole *via* ATG-dependent macroautophagy (Yamagata et al., 2011; Tsuji et al., 2017). Several recent reports demonstrated the existence of microdomains in the plasma membrane in plants (Klima and Foissner, 2008; Furt et al., 2010; Cacas et al., 2012), and proteomic approaches showed the existence of microdomains also in the tonoplast (Ozolina et al., 2013; Yoshida et al., 2013). However, the cell biological significance and function of microdomains in the plant tonoplast need to be clarified. Although the results shown here were obtained in the presence of an inhibitor and it is necessary to verify these data in future analyses, this report may support the existence of microdomains in the tonoplasts in plants and indicates some biological significance; starvation induces microautophagy and the invagination may occur at the microdomain in the tonoplast.

### What Does Microautophagy Target Under Starvation?

The identity of E-64d vesicles has not been fully elucidated. However, even in cells not treated with E-64d, FM-4-64–labeled vesicles are formed under starvation (**Figure 7A**, mock and +ConA). The formation of vesicles by the addition of E-64d, under conditions of starvation, has been detected by bright field observation (Inoue et al., 2006). In other words, the vesicle formation under the treatment of E-64d and FM4-64 is unlikely to contradict the natural intracellular phenomenon. As mentioned above, the amount of tonoplast was decreased after the induction of sucrose starvation (**Figure 4A**). This strongly suggested the existence of microautophagy digesting the vacuole membrane, since the tonoplast is composed of lipids and can be a carbon source for plant cells to survive under starvation. In addition, the cytosolic acid granules were captured in the E-64d vesicles (**Figure 5**), the contents of which could be acid phosphatases as detected by electron microscopy (Moriyasu and Ohsumi, 1996). Lysosomes in mammalian cells accumulate acid phosphatases, and the enzyme becomes functional when endosomes fuse to the lysosomes. In tobacco BY-2 cells, the accumulation of acidic compartment under starvation implicates that plant cells increase degradation activity in response to starvation.

FM4-64 can visualize not only the vacuolar membrane, but also the plasma membrane and endosomes. Although we use cells that visualize only the vacuolar membrane in our detailed analysis (**Figures 5**–**7**), it was necessary to discuss the possibility that the cell membrane and endosomes formed the vesicles. BFA is an inhibitor of endomembrane trafficking, and is known to induce TGN and endosome aggregation, which is called as a BFA body, in *Arabidopsis* root and cotyledon cells (Robinson et al., 2008). The aggregates of E-64d vesicles are similar to the BFA body. However, we demonstrated that the endosomal markers GFP-SYP43 and GFP-ARA7 signals are not detected on the E-64d vesicles (**Supplementary Figure 9**), indicating that the aggregates of E-64d vesicles are not identical with BFA body. On the other hand, presence or absence of endosomal components in E-64d vesicles should be discussed carefully. It is suggested that endocytosis, vacuolar transport, and autophagy pathways are not completely separated and share part of the pathways (Fan et al., 2015). In addition, the direct uptake of cytosolic components into the MVB and their degradation in the vacuole, called endosomal microautophagy, is reported (Sahu et al., 2011; Mejlvang et al., 2018). PIP2a is a plasma membrane protein, accumulated at the E-64d vesicles under starvation, and PIP2a degradation was inhibited by the treatment of E-64d (Yamada et al., 2005). Therefore, further studies are needed to clarify the relationship between endosomes and tonoplast-derived vesicles.

In summary, starvation induces microautophagy to recycle cellular components; the tonoplast invaginates toward the vacuolar lumen and captures the cytosolic compartment, including acid granules and autophagosomes, and forms vesicles to compartmentalize the degrading components. During this process, ATG2, ATG5, ATG7, and partially ATG8a are involved in the vesicle formation, although it is still unclear how these proteins contribute to the process of starvation-induced microautophagy. The vesicles may be formed at the microdomain in which the tonoplast protein is excluded, and the vesicles are released into the vacuole to be degraded. On the other hand, E-64d inhibits the degradation of vesicles and accumulates them to the tonoplast. Further studies will be required in order to understand the detailed machinery of microautophagy in plants.

## Data Availability Statement

Our NGS data has been submitted to a public database DDBJ (https://www.ddbj.nig.ac.jp). The accession number is DRA009208. 

## Author Contributions

SG-Y and KeY conceived and designed the experiments. SG-Y, KeY, and JB performed most of the experiments. KO, SM, and MN contributed to mutant screening and phenotyping. SS, KaY, KO, and MH contributed to the next-generation sequencing. HU and IH-N contributed to production of the Venus-VAM3 transgenic plant. SG-Y and KeY analyzed the data and wrote the article.

## Funding

This study was supported by the National Science Centre, Poland [UMO-2016/21/P/NZ9/01089 to SG-Y (the project has received funding from the European Union’s Horizon 2020 research and innovation program under the Marie Skłodowska-Curie grant agreement no. 665778) and UMO-2016/23/B/NZ1/01847 to KeY]; the Foundation for Polish Science (TEAM/2017-4/41 to KeY); KAKENHI from the Japan Society for the Promotion of Science, Japan (JP15J40032 to SG-Y, JP17K07457 to SM, and JP15H05776 to IH-N); and KAKENHI from the Ministry of Education, Culture, Sports, Science and Technology, Japan (JP26111523 to SG-Y); as well as the institutional support provided from the National Institute for Basic Biology (NIBB), Kyoto University, and Małopolska Centre of Biotechnology, Jagiellonian University. Next-generation sequencing was supported by NIBB Collaborative Research Programs 11-711.

## Conflict of Interest

The authors declare that the research was conducted in the absence of any commercial or financial relationships that could be construed as a potential conflict of interest.
