# Supplementary material for: Sucrose Starvation Induces Microautophagy in Plant Root Cells
Source: Front Plant Sci. 2019 Dec 3;10:1604. doi: 10.3389/fpls.2019.01604 (PMC6901504; doi:10.3389/fpls.2019.01604)
Supplement: Supplementary file 1 [file DataSheet_1.pdf]

SUPPLEMENTARY MATERIAL

Supplementary Figure 1. Peroxisome phenotypes in WT, *peup17* and *peup22*.

Supplementary Figure 2. Peroxisome localization in the FM4-64- and E-64d-treated starved cells.

Supplementary Figure 3. The phenotype of E-64d vesicle formation in the *ap2m-2* and *ap2s-1* mutants.

Supplementary Figure 4. Allelism test between *peup17* and *atg5-1*, and *peup22* and *atg7-2*.

Supplementary Figure 5. The phenotypes of E-64d vesicles in WT, *peup17* and *peup22*.

Supplementary Figure 6. A graph representing the number of acidic granules in BY-2 cells under starvation.

Supplementary Figure 7. The detailed images of root cells in WT, *atg5-1* and *atg7-2*.

Supplementary Figure 8. Simultaneous treatment of E-64d and ConA to the Arabidopsis root cells.

Supplementary Figure 9. Subcellular localization of mGFP-VAMP713, GFP-SYP43 and GFP-ARA7 in the FM4-64- and E-64d-treated starved cells.

Supplementary Figure 10. Snapshot images from the time-lapse movie Supplementary Movie 6.

Supplementary Movie 1. A 3D image of the Venus-VAM3 root cell after 5 h of the E-64d treatment and starvation.

Supplementary Movie 2. A time-lapse movie of releasing an E-64d vesicle from the tonoplast (10X speed).

Supplementary Movie 3. A time-lapse movie of the motions of E-64d vesicles in the Venus-VAM3 (1X speed).

Supplementary Movie 4. A time-lapse movie of the motions of E-64d vesicle s in the *atg5-1* (1X speed).

Supplementary Movie 5. A time-lapse movie of the motions of E-64d vesicles in the *atg7-2* (1X speed).

Supplementary Movie 6. A time-lapse movie of the capturing of an ATG8a-containing vesicle (10X speed).

Supplementary Movie 7. A time-lapse movie of the vanishing GFP fluorescence in an E-64d vesicle (10X speed)

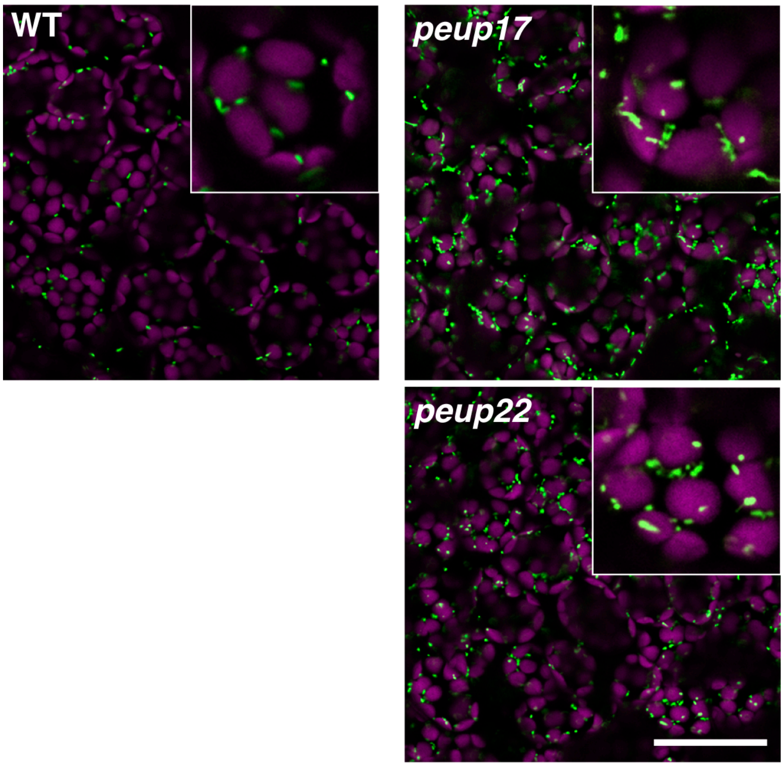

Supplementary Figure 1. GFP fluorescence patterns of the wild-type (WT) and the *peup17* and *peup22* mutants, which express the peroxisome marker GFP-PTS1. Leaves of 2-week-old plants were examined using confocal laser-scanning microscopy. Green indicates GFP signals and magenta indicates autofluorescence from chloroplasts. Bar = 50 µm and the boxes of the magnified images are 30 µm.

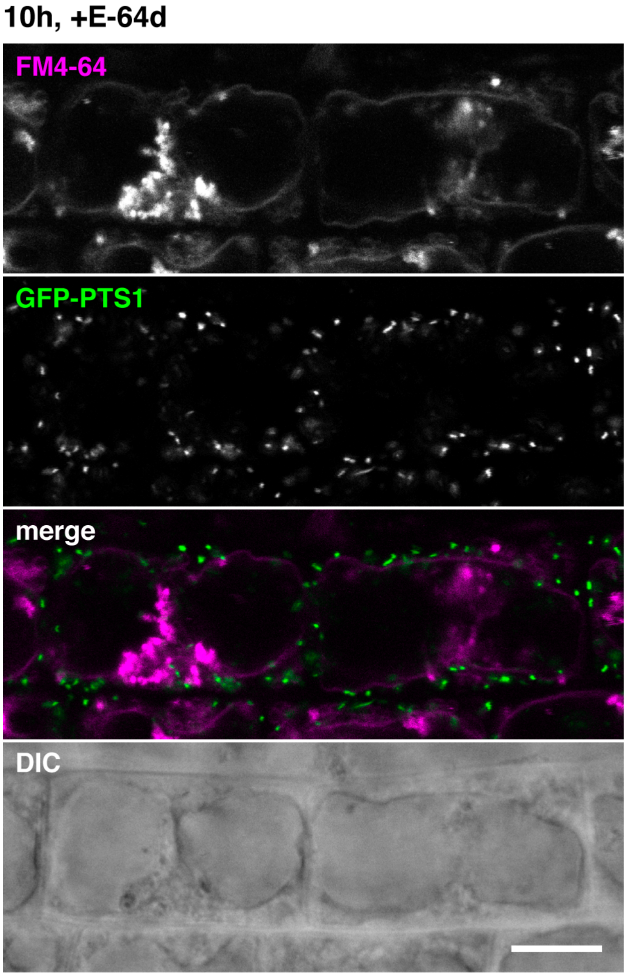

Supplementary Figure 2. Subcellular localization of peroxisomes during starvation and E-64d treatment. (A) Confocal micrographs of root cells from GFP-PTS1 transgenic plants treated with E-64d under sucrose starvation. The tonoplast was visualized with FM4-64 before the induction of starvation. Cells were starved and treated with E-64d for 10 h. Magenta and green indicate the signals of FM4-64 and GFP, respectively. Bar = 10 µm.

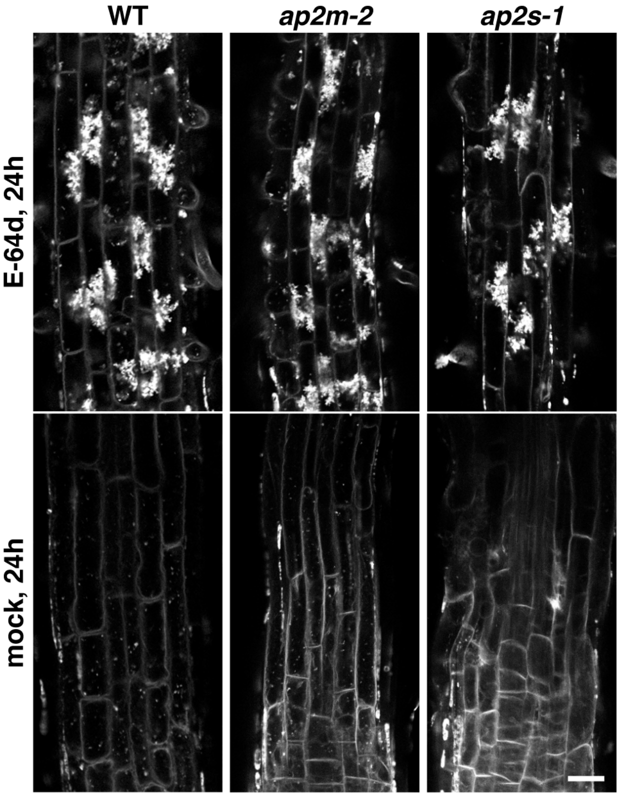

Supplementary Figure 3. The phenotype of E-64d vesicle formation in the root of WT, *ap2m-2* and *ap2s-1* mutants. Six-day-old seedlings were were stained with FM4-64 with or without E-64d for 24 h. Bar = 20 µm.

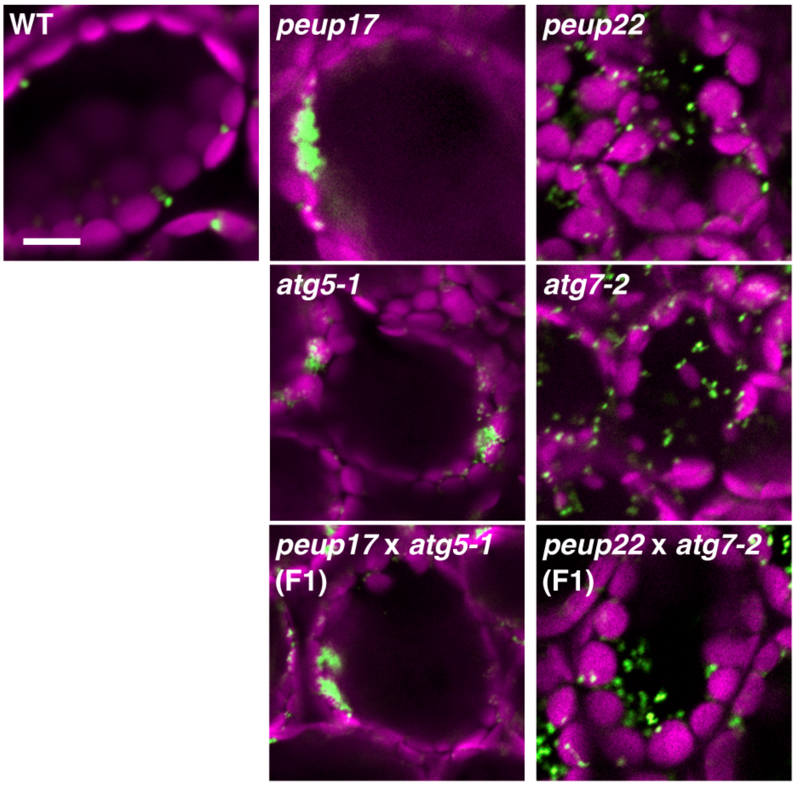

Supplementary Figure 4. Allelism test between *peup17* and *atg5-1*, and *peup22* and *atg7-2*. The phenotypes were determined with the peroxisome marker GFP-PTS1 (green). Magenta, autofluorescence from chloroplasts. Bar = 10 µm.

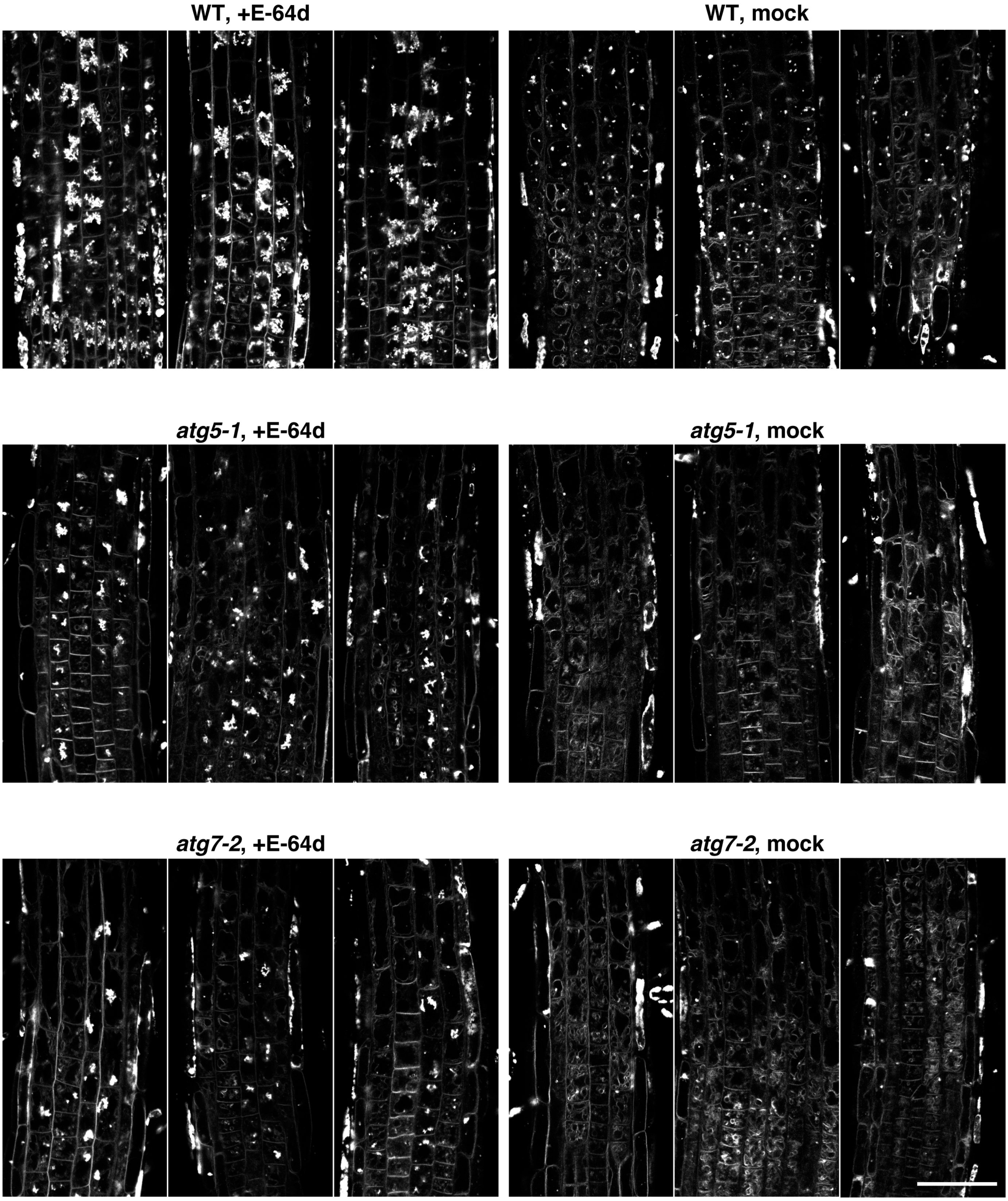

Supplementary Figure 5. Confocal microscopic images of wild-type (WT) and the *atg* mutants. The plats were treated with or without E-64d for 24 hours under sucrose starvation. Membranes were stained with FM4-64. Three roots were taken pictures in each line. Bar = 50 µm.

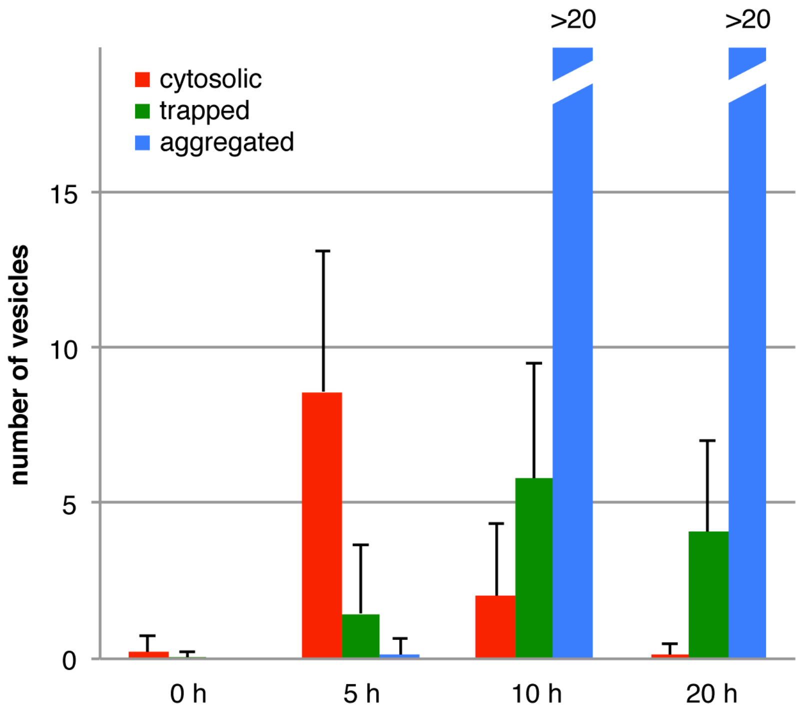

Supplementary Figure 6. A graph representing the number of acidic granules in BY-2 cells under starvation. The number of the granules in the cytosol, trapped in the invagination and aggregated were counted at the indicated time after the induction of starvation and E-64d treatment. More than 15 cells were used at each time point. Bar = ± SD.

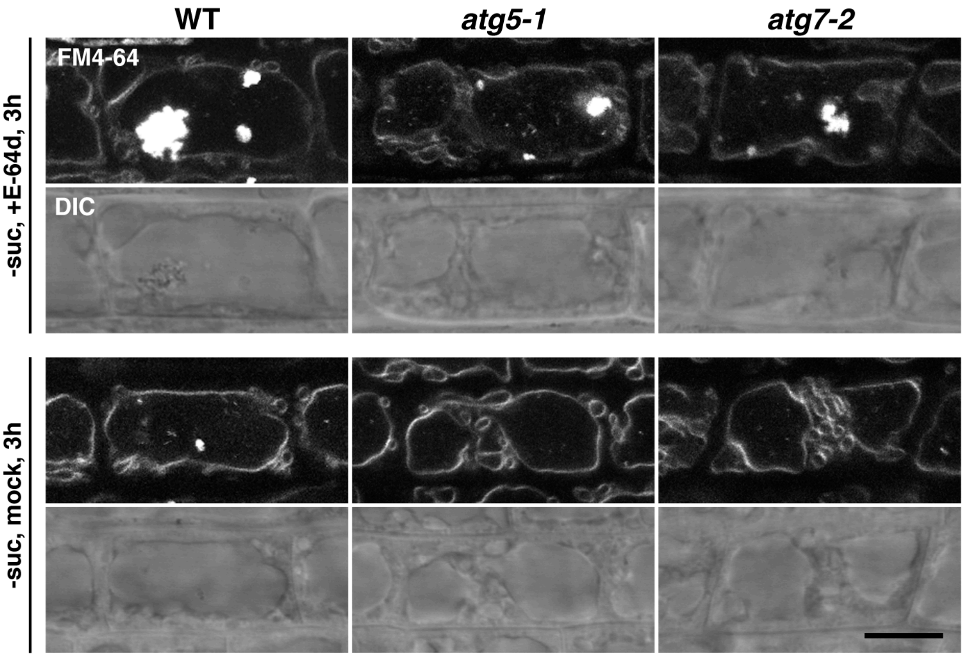

Supplementary Figure 7. Fluorescence and DIC images of root cells of elongation zone in WT, *atg5-1* and *atg7-2*. The tonoplasts were pre-stained with FM4-64 and then cells were starved with the E-64d/mock treatment for 3 h. Bar = 10 µm.

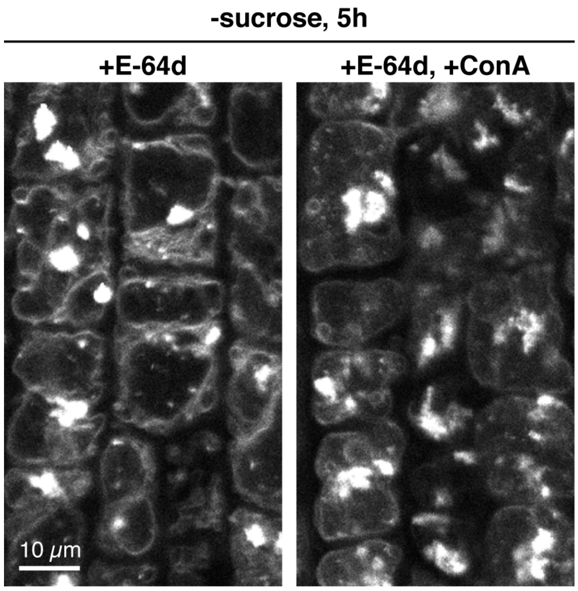

Supplementary Figure 8. Simultaneous treatment of E-64d and ConA to the Arabidopsis root cells. The tonoplasts were pre-stained with FM4-64, and then cells were starved with the simultaneous treatment of E-64d and ConA for 5 h.

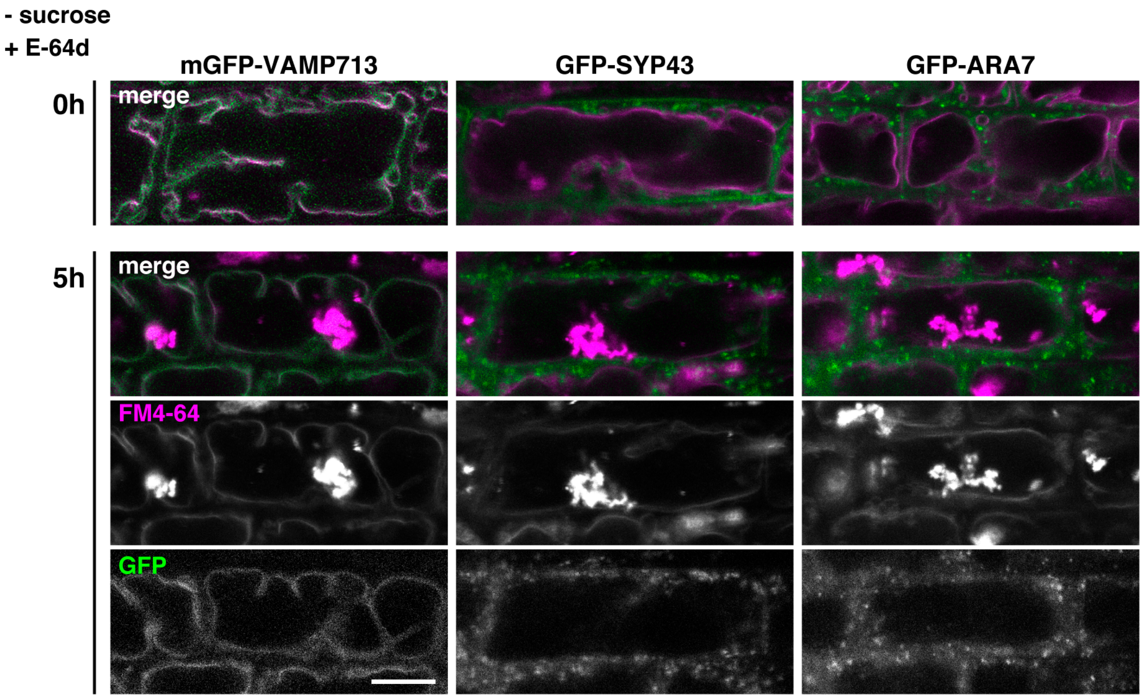

Supplementary Figure 9. Subcellular localizations of mGFP-VAMP713, GFP-SYP43 and GFP-ARA7. The tonoplasts were pre-stained with FM4-64 (0 h) and induced starvation with the E-64d treatment for 5 h. Bar = 10 μm.

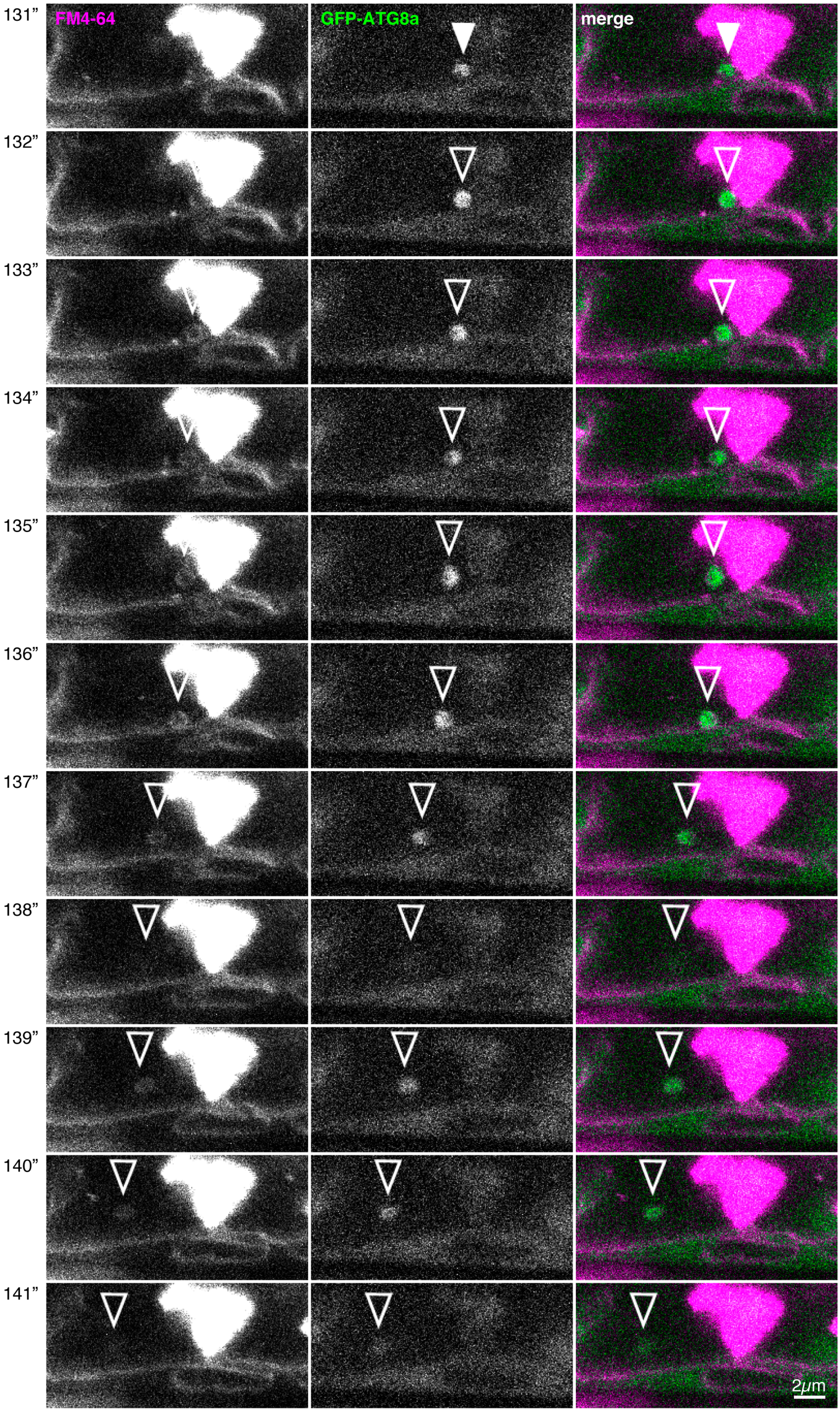

131”
132”
133”
134”
135”
136”
137”
138”
139”
140”
141”
FM4-64
GFP-ATG8a
merge
2μm

Supplementary Figure 10. Time-lapse images of capturing autophagosomes by the tonoplast. The images were extracted from the Supplementary Movie 6. The tonoplast was pre-stained with FM4-64, and then cells were starved with E-64d treatment for 3 h. A white arrowhead indicates captured autophagosome, and black arrowheads indicate the vesicles which started random motion in the vacuole. The numbers at the left side indicate the elapsed time.

Supplementary Table 1. Nucleotide Sequences of Primers for RT-PCR in Figure 2.

Primer name	Sequence	Note
#1	5'-ATGGCGAAGGAAGCGGTCA-3'	ATG5 Forward primer binding at the start ATG.
#2	5'-TGGTCTTTCGGGTTCTGCAC-3'	ATG5 Reverse primer binding at 3rd exon.
#3	5'-CAGAATCTTCTCCTTCACATGG-3'	ATG5 Reverse primer binding at 4th exon.
#4	5'-CCATGTGAAGGAGAAGATTCTG-3'	ATG5 Forward primer binding at 4th exon.
#5	5'-TCACCTTTGAGGAGCTTTCAC-3'	ATG5 Reverse primer binding at the stop codon.
#6	5'-ATGGCTGAGAAAGAAACTCCA-3'	ATG7 Forward primer binding at the start ATG.
#7	5'-TTAAAGATCTACAGCTACATCGT-3'	ATG7 Reverse primer binding at the stop codon.
UBQ10_Q2_F	5'-GAAGTGGAAAGCTCCGACAC-3'	Primer for UBQ10 gene, internal control of gene expression.
UBQ10_Q2_R	5'-TTAGAAACCACCACGAAGACG-3'	Primer for UBQ10 gene, internal control of gene expression.
